# Limiting Molecular Twisting: Upgrading a Donor–Acceptor Dye to Drive H_2_ Evolution

**DOI:** 10.1002/advs.202403454

**Published:** 2024-08-26

**Authors:** Kaijian Zhu, Ainoa Paradelo Rodríguez, Maria B. Brands, Titus de Haas, Francesco Buda, Joost N.H. Reek, Guido Mul, Annemarie Huijser

**Affiliations:** ^1^ PhotoCatalytic Synthesis Group MESA+ Institute for Nanotechnology University of Twente P.O. Box 217 Enschede 7500 AE The Netherlands; ^2^ van ‘t Hoff Institute for Molecular Sciences University of Amsterdam Science Park 904 Amsterdam 1098 XH The Netherlands; ^3^ Leiden Institute of Chemistry Leiden University P.O. Box 9502 Leiden 2300 RA The Netherlands

**Keywords:** donor–acceptor dye, dye‐sensitized photocathode, H2 evolution, molecular twisting, TICT

## Abstract

The donor–acceptor (D–A) dye 4‐(bis‐4‐(5‐(2,2‐dicyano‐vinyl)‐thiophene‐2‐yl)‐phenyl‐amino)‐benzoic acid (P1) has been frequently used to functionalize NiO photocathodes and induce photoelectrochemical reduction of protons when coupled to a suitable catalyst. Photoinduced twisting of the P1 dye is steered on NiO by co‐adsorption of tetradecanoic acid (C_14_, myristic acid (MA)). Density Functional Theory and time‐resolved photoluminescence studies confirm that twisting lowers the energy levels of the photoexcited D–A dye. The apolar environment provided by the MA suppresses photoinduced D–A twisting, retards charge recombination following photoinduced charge separation between P1 and NiO, and provides a larger electrochemical potential increasing the photocurrent. Very interestingly, co‐adsorption of MA induces H_2_ evolution upon photoexcitation without the presence of an H_2_ evolution catalyst. Based on prior art, the formation of H_2_ is assigned to the dissolution of Ni^2+^, followed by reduction and re‐deposition of Ni nanoparticles acting as the catalytically active site. It propose that only excited P1 with suppressed twisting provides the sufficient electrochemical potential to induce deposition of Ni nanoparticles. The work illustrates the importance of understanding the effects of photoinduced intramolecular twisting and highlights the promise of designing twisting‐limited D–A dyes to create efficient solar fuel devices.

## Graphical abstract

Mysteric acid co‐adsorbed with the P1 dye onto NiO provides a local apolar environment and reduces photoinduced donor–acceptor twisting of the dye. The resulting larger electrochemical potential enables H_2_ evolution even in the absence of a proton reduction catalyst.

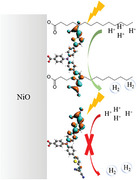

## 1 Introduction

Dye‐sensitized photoelectrochemical cells (DSPECs) have attracted wide attention to converting solar energy into high‐energy chemicals such as hydrogen.^[^ ^1^, ^2^, ^3^, ^4^ ^]^ A dye‐sensitized electrode typically consists of a dye molecule immobilized on an oxide semiconductor, linked to an appropriate catalyst for hydrogen evolution (cathode) or oxygen evolution (anode). When used as a photocathode, a commonly used dye is P1 [4‐(bis‐4‐(5‐(2,2‐dicyano‐vinyl)‐thiophene‐2‐yl)‐phenyl‐amino)‐benzoic acid] often immobilized on a p‐type semiconductor like NiO. Suppression of ultrafast charge recombination is a core challenge to achieve an efficient photocathode, which typically limits the performance of tandem DSPECs.^[^ ^5^, ^6^, ^7^ ^]^ Strategies such as doping of the semiconductor^[^ ^8^, ^9^ ^]^ and designing novel dyes and catalysts^[^ ^10^, ^11^, ^12^, ^13^ ^]^ have therefore been extensively explored. The development of push‐pull dyes with an electron donor–*π* bridge–electron acceptor (D–A) structure is one of the most important breakthroughs to retard charge recombination. The highest occupied molecular orbital (HOMO) and the lowest unoccupied molecular orbital (LUMO) are usually located at different parts of the D–A structure.^[^ ^10^, ^14^, ^15^ ^]^ In this way the photoinduced electron and hole are spatially separated, enabling longer‐lived charge separation and a higher device performance.

**Figure 1:**
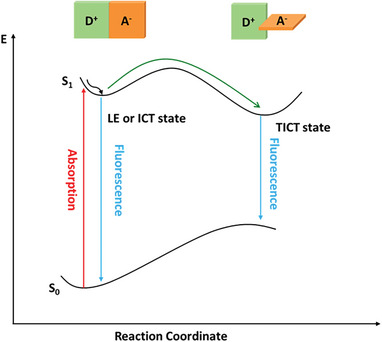
Potential energy diagram for photoinduced twisting of a donor–acceptor (D–A) dye.

Due to the spatial separation of the electron and hole, the dipole moment of the dye changes after photoexcitation. To stabilize the excited molecular complex, twisting between donor and acceptor typically occurs following photoexcitation. This process results in a so‐called twisted intramolecular charge transfer (TICT) state, a concept established in 1973^[^ ^16^ ^]^ and relatively common in D–A molecules (**Figure** **1**).^[^ ^17^, ^18^ ^]^ The TICT mechanism has guided the design of molecular complexes for different applications, such as sensing^[^ ^19^ ^]^ or fluorescent probes.^[^ ^20^, ^21^ ^]^ The internal conversion from the locally excited (LE) or intramolecular charge transfer (ICT) state into the TICT state is a non‐radiative process, which can quench the fluorescence of the LE/ICT state.^[^ ^22^, ^23^ ^]^ Though D–A dyes are widely applied in dye‐sensitized solar cells and photoelectrochemical cells, only a few studies focus on the potential impact of photoinduced twisting on the performance. Such studies have solely been performed for dye‐sensitized solar cells and have never been correlated to photochemical conversion, and the conclusions are contradictory. The latter is likely caused by the approaches used: twisting has been controlled by structural modification of the dye, potentially also affecting its electronic properties, rather than our strategy of keeping the dye unaffected and suppressing TICT by co‐adsorption of MA on the NiO surface. According to Ghosh and co‐workers, twisting reduces charge recombination and increases the performance of solar cells.^[^ ^24^ ^]^ This conclusion is in line with studies by Karlsson et al.^[^ ^25^ ^]^ and Sun and colleagues,^[^ ^26^ ^]^ who observed that promoting TICT gives a better performance. In contrast, Kwon et al. reported that a twisted dye shows a threefold lower power conversion efficiency relative to the planar analogue due to slower light‐induced intramolecular charge transfer.^[^ ^27^ ^]^ As the molecular complexes were modified either by alkyl cyclization or by adding an additional donor to achieve or inhibit twisting, a comparison of these results is not straightforward. The polarity of the environment can have a significant impact, with a polar environment typically favoring twisting and apolar conditions inhibiting twisting.^[^ ^17^ ^]^ Also, Goodson and co‐workers observed superior performance for a complex with a planar acceptor relative to a twisted acceptor.^[^ ^28^ ^]^

In this work, the benchmark P1 dye (4‐(bis‐4‐(5‐(2,2‐dicyano‐vinyl)‐thiophene‐2‐yl)‐phenyl‐amino)‐benzoic acid), designed for photosensitization of p‐type semiconductors, has been applied as the light absorber. The P1 dye has a carboxylic anchoring group, a triphenylamine moiety as the electron donor, a thiophene‐based bridge, and an electron‐accepting malononitrile moiety.^[^ ^14^, ^29^ ^]^ The effect of enabled or inhibited TICT on the photodynamics and performance of a dye‐sensitized photocathode is studied by co‐adsorption of P1 and myristic acid (MA, 1‐tridecanecarboxylic acid) onto nanoporous NiO. MA has the same carboxylic acid anchoring group as P1 and provides a local apolar environment. Time‐resolved photoluminescence and Density Functional Theory (DFT) studies show that TICT decreases the reduction potential of photoexcited electrons, while MA suppresses twisting. Very interestingly, in the presence of MA light‐induced H_2_ evolution is observed in aqueous media, despite the absence of a H_2_ evolution catalyst. Our work demonstrates the promise of designing TICT‐controlled dyes to realize highly efficient photocathodes.

## 2 Results and Discussion

### 2.1 Nanostructure Characterization and Steady‐State Absorption Spectra

**Figure 2:**
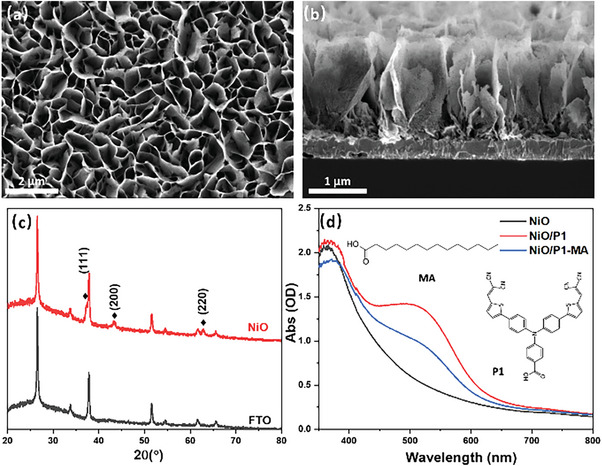
a) Surface and b) cross‐sectional SEM images, c) XRD patterns of FTO and NiO/FTO, and the peak assignments for NiO and d) UV–vis absorbance spectra of NiO before and after P1 sensitization in the absence and presence of 40 mm MA in the solution used for sensitization. The structure of the P1 dye and MA are shown in the inset of (d).

The surface and cross‐section scanning electron microscopy (SEM) micrographs of NiO on FTO presented in **Figures** **2**a,b show a leaf‐like porous film structure with a thickness of ≈1.8 µm. The X‐ray diffraction (XRD) patterns of NiO/FTO and FTO shown in Figure 2c agree with literature data,^[^ ^9^ ^]^ which confirms the film consists of NiO and does not have significant impurities. Figure 2d shows the UV–vis absorbance spectra of NiO/FTO corrected for the FTO substrate, NiO films sensitized with only P1, and with both P1 and MA. The weak absorbance of NiO in the visible is likely due to trap states,^[^ ^30^, ^31^, ^32^ ^]^ while the steep rise <380 nm is in agreement with the bandgap of 3.4–4.3 eV.^[^ ^33^ ^]^ The P1 dye is hence predominantly responsible for the visible light absorption.^[^ ^14^, ^29^ ^]^ DFT calculations indicate that in the equilibrium geometry, i.e., the structure before photoexcitation, the HOMO is mostly localized on the donor part of the molecule, while the LUMO is mainly localized on the acceptor part.^[^ ^14^ ^]^ P1 shows absorption bands ≈300–420 nm and 400–700 nm, assigned to *π*‐*π* ^*^ transitions.^[^ ^34^ ^]^ A broadening and red‐shift in absorption spectra are typically observed when P1 is adsorbed on NiO, likely due to electronic coupling between P1 and NiO and deprotonation of the anchoring carboxylic acid group.^[^ ^12^, ^35^ ^]^ The presence of MA in the P1 solution used for NiO photosensitization lowers the absorption in the visible, likely due to competitive adsorption on the NiO surface, reducing the quantity of surface‐bound P1 molecules. Indeed, the surface coverage of the P1 dye on the NiO surface is reduced to ≈48% and the light absorption efficiency is decreased by ≈25% when MA is present (Figure S1, Supporting Information). Except for a reduction in absorbance (Figure 2d; Figures S1a,b, Supporting Information), no spectral changes are observed (Figure S1c, Supporting Information), showing that MA neither affects the electronic levels of the P1 dye nor the concentration of Ni^3+^ in NiO, since the latter would give rise to visible absorption ≈600–700 nm.^[^ ^30^ ^]^ The Raman spectra do not show obvious differences in the absence or presence of co‐adsorbed MA (Figure S2, Supporting Information), indicating that NiO‐dye interactions are similar in both cases.

### 2.2 Effect of Reduced Twisting on the Photodynamics

**Figure 3:**
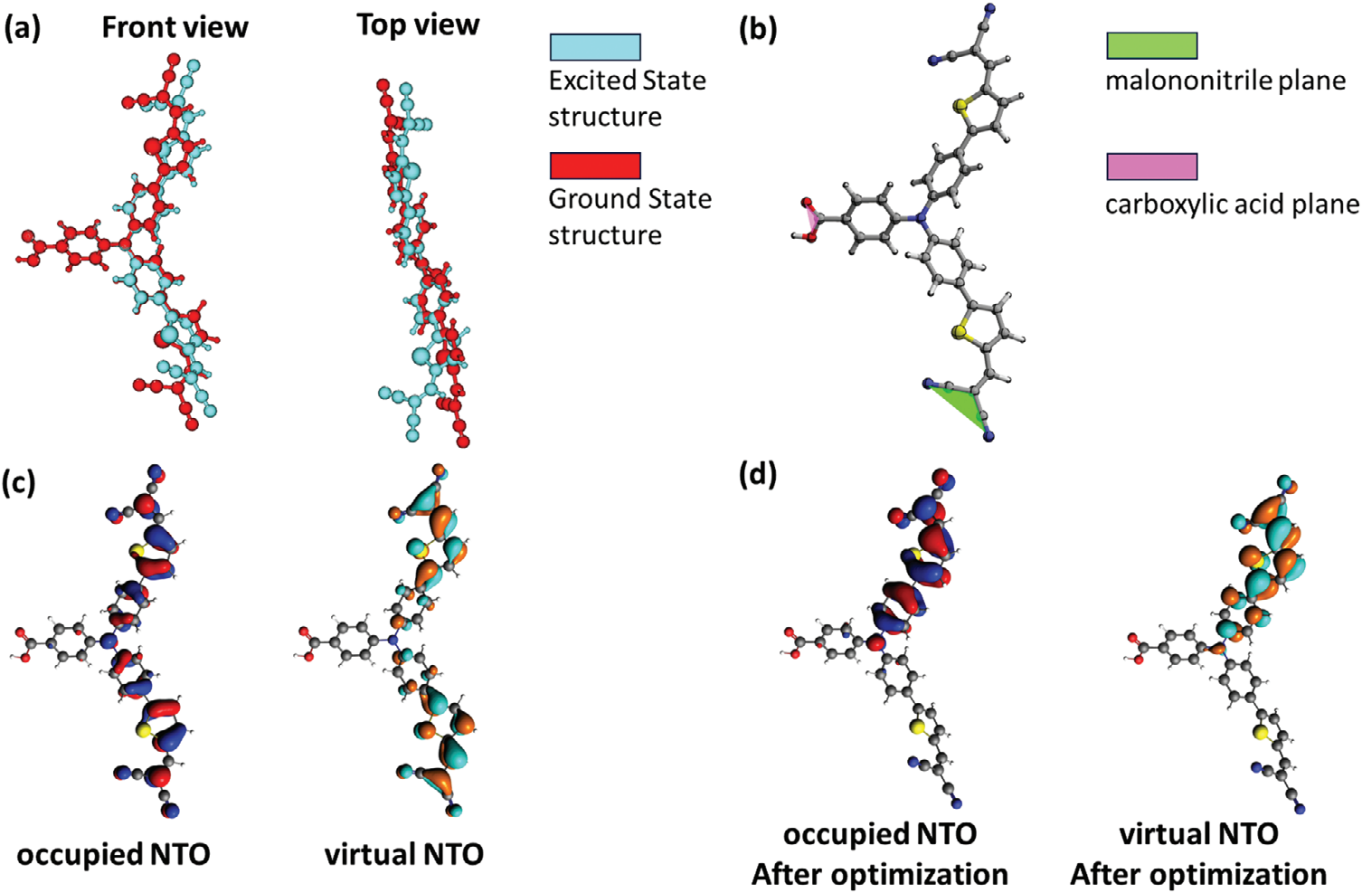
Structural rearrangement of P1 after excitation a). The ground state‐optimized structure is visualized in red, while the excited state‐optimized structure is depicted in cyan. The malononitrile and carboxylic acid planes that capture the geometric rearrangement after excitation are highlighted in green and purple, respectively b). The occupied and virtual natural transition orbitals (NTOs) of the S_0_ to S_1_ excitation in the structure optimized in the ground state c) and the excited state d).

**Figure** **3**a shows the structural rearrangement of the free P1 dye after excitation, calculated with Time‐Dependent Density Functional Theory (TDDFT). In the ground state, the S_0_ to S_1_ excitation was found to contain 69.6% HOMO‐LUMO transition character, while the HOMO‐1 to LUMO+1 excitation contributed 19.9%. After the excited state geometry relaxation, the HOMO‐LUMO contribution increased to 85.7%, while the HOMO‐1 to LUMO+1 contribution decreased to 1.8%. In order to visualize the transition as localized excitation, we calculated the natural transition orbitals (NTOs).^[^ ^36^ ^]^ Figure 3c shows the occupied and virtual NTOs associated with the S_0_ to S_1_ excitation in the optimized ground state structure. Visualizations of the HOMO‐1, HOMO, LUMO, and LUMO‐1 orbitals are provided in Figure S3 (Supporting Information). Interestingly, the NTOs localize more on the tails of the P1 dye than the HOMO and LUMO orbitals. After excitation, the TDDFT excited state optimization shows that the P1 molecule undergoes a structural change, breaking the pseudo‐C_2_ symmetry around the C─C bond axis connecting the carboxyl and the phenyl, by twisting one of the two electron‐accepting tails. To quantify the degree of twisting, the angle between the plane of the malononitrile and the carboxylic acid anchoring group was tracked during the optimization (see Figure S4, Supporting Information). It was found that this angle increases by 46.0° from the ground state optimized structure to the excited state optimized structure (from 47.9° to 93.9°). After this twisting, the occupied and virtual NTOs are now located entirely on the non‐twisted tails (Figure 3d), resulting in a stabilization of the excited state by 0.36 eV with respect to the Franck–Condon point and an overall decrease in the S_0_ to S_1_ transition energy of 0.61 eV. The NTOs show that the S_0_ to S_1_ excitation involves charge density transfer from the triphenylamine core to the malononitrile, right after excitation. Then, after the structural rearrangement, the two NTOs involved are spatially more overlapping. Nevertheless, the oscillator strength for the S_0_ to S_1_ excitation for the twisted dye is lower than for the non‐twisted dye (2.57 vs 1.89). Because the hole injection from the dye to the NiO surface (typically biphasic, either in <100–150 fs or in 1–20 ps^[^ ^38^ ^]^) is likely fast compared to the twisting timescale, an additional structure optimization was performed on the reduced dye (P1^•−^). It was found that the twisting was largely preserved, with the angle between the carboxylic acid plane and the plane of the twisted malononitrile twisting 33.6° with respect to the ground state optimized structure (from 47.9° to 81.5°) (Figure S5, Supporting Information). The symmetry breaking in the excited state and reduced state has also been reported by Gibson et al., who found qualitatively similar results.^[^ ^37^ ^]^

**Figure 4:**
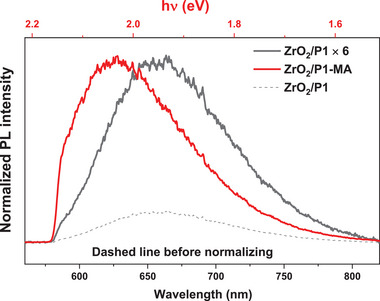
Normalized steady‐state photoluminescence spectra of ZrO_2_/P1 and ZrO_2_/P1‐MA in air recorded using 532 nm excitation, detected using a 580 nm long‐pass filter to remove residual excitation light.

As the photoluminescence (PL) from the TICT state can be expected to be red‐shifted compared to the LE/ICT state (Figure 1 and DFT calculations discussed above), light‐induced P1 twisting in the absence and presence of co‐adsorbed MA has been studied by PL spectroscopy. Due to fast hole injection from the excited P1 dye (P1^*^) into the NiO (typically biphasic, either in <100–150 fs or in 1–20 ps^[^ ^38^ ^]^), NiO/P1 and NiO/P1‐MA both show a very low PL intensity (Figure S6, Supporting Information), likely originating from a tiny amount of non‐injecting dye molecules and therefore not representative for comparison. To investigate the impact of co‐adsorbed MA on the photoinduced twisting process, ZrO_2_ was therefore used as supporting oxide instead of NiO. The steady‐state PL spectra shown in **Figure** **4** demonstrate a ≈0.09 eV blue‐shift for ZrO_2_/P1‐MA relative to ZrO_2_/P1, indicating that co‐adsorbed MA suppresses twisting. The absence of a shift in PL spectrum when reducing the dye loading of P1/ZrO_2_ (Figure S7, Supporting Information) excludes an effect of MA on potential P1 aggregation. The PL intensity of ZrO_2_/P1‐MA exceeds that of ZrO_2_/P1 by a factor of six, despite the lower P1 loading and light absorption, in agreement with earlier studies showing that twisting reduces the PL quantum yield.^[^ ^39^, ^40^ ^]^ The absence of a blue‐shift and lower PL intensity for a Ru‐dye without D–A structure (Figures S9 and S10, Supporting Information) in the presence of MA consolidates this interpretation. The PL spectra of ZrO_2_/P1 samples prepared from P1 solutions with different MA concentrations show an increasing blueshift with MA concentration (Figure S8, Supporting Information), providing additional evidence for a reduction in P1 twisting by MA, and suggesting a correlation between the average twisting angle and the quantity of MA molecules. Also, co‐adsorbed nonanoic acid (shorter apolar alkyl chain than MA) and 2,5,8,11‐tetraoxatridecan‐13‐oic acid (long polar instead of apolar chain) do not show the same ability to reduce twisting as MA (Figure S11, Supporting Information). The effect of MA on the NiO/P1 interface photodynamics is hence predominantly due to the apolar environment, and less due to steric hindrance. Maffeis et al. measured the PL spectra of P1 in different solvents,^[^ ^35^ ^]^ and observed a blue‐shift in PL in the order acetonitrile, tetrahydrofuran, and toluene, possibly due to inhibited molecular twisting in an apolar solvent. Except for the solvent polarity, the solvent viscosity can also affect the molecular twisting dynamics, therefore, we measured the steady‐state and time‐resolved PL of ZrO_2_/P1 in two protic solvents, methanol, and glycerol, which have different viscosity (Figure S12, Supporting Information). It is obvious that in glycerol, i.e., a higher viscosity, the PL spectra also show a blueshift and higher intensity as compared to ZrO_2_/P1‐MA and ZrO_2_/P1 (Figure S12c, Supporting Information). Based on these results, the blue‐shift in PL is assigned to limited P1 twisting in the presence of MA, and a significant effect on dye aggregation affecting the PL spectrum can be excluded.

**Figure 5:**
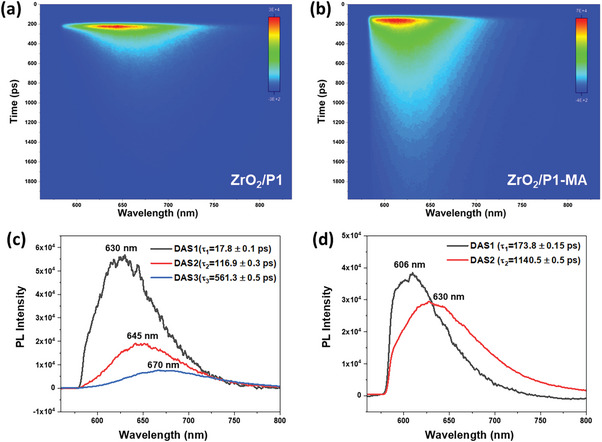
a) Time‐resolved photoluminescence spectral profiles of ZrO_2_/P1 and b) ZrO_2_/P1‐MA in air after 532 nm excitation, detected using a 580 nm long‐pass filter, and the decay associated spectra (DAS) and lifetimes from global analysis using a parallel decay model for c) ZrO_2_/P1 and d) ZrO_2_/P1‐MA.

The twisting process in the absence and presence of co‐adsorbed MA has been further investigated by time‐resolved photoluminescence (TRPL, **Figure** **5**). Albeit the spectra of ZrO_2_/P1 a) are red‐shifted relative to those of ZrO_2_/P1‐MA b), both show a red‐shift in time following excitation. The co‐adsorbed MA prolongs the PL decay (Figure S13, Supporting Information), which shows that photoinduced charge transfer from P1 to MA is unlikely. The TRPL data are well described by a parallel model (Figure S14, Supporting Information), and the temporal profiles and fits are shown in Figure S15 (Supporting Information). The obtained decay‐associated spectra (DAS) and the corresponding lifetimes are shown in Figures 5c (ZrO_2_/P1) and 5d (ZrO_2_/P1‐MA). Four DAS can be identified: centered at 606 nm (only for ZrO_2_/P1‐MA), 630 nm (for ZrO_2_/P1 and ZrO_2_/P1‐MA), and at 645 and 670 nm (only for ZrO_2_/P1). Considering the suppressed P1^*^ twisting by MA, the dependency of the PL shift on the MA quantity (Figure S8, Supporting Information) and the similar UV–vis spectra in absence and presence of MA (Figure S1, Supporting Information), we assign the PL bands centered at 606, 630, 645, and 670 nm to a little (606 nm), two partly (630 and 645 nm) and a fully twisted TICT state (670 nm) (Figure S14, Supporting Information). The latter two states are only observed in case MA is absent, while in that case the 606 nm band, assigned to the only slightly twisted structure relative to the LE/ICT state, cannot be identified any longer. Femtosecond transient absorption (TA) studies indicate that (slight, partial, or full) twisting occurs in ≈17 ps for ZrO_2_/P1 and ≈35 ps for ZrO_2_/P1‐MA (Figures S14–S16, Supporting Information), quenching the fluorescence of the LE/ICT state.

**Figure 6:**
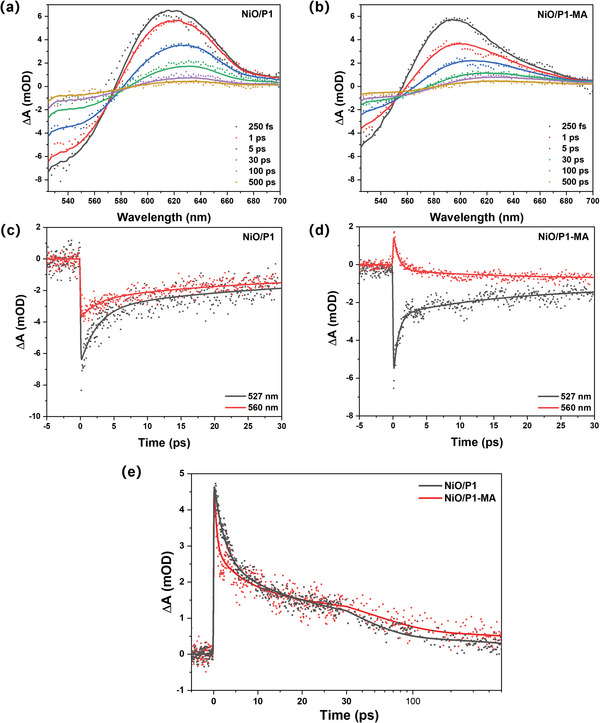
Transient absorption spectra after excitation at 500 nm a,b) and kinetic traces c,d) of NiO/P1 (a,c) and NiO/P1‐MA (b,d) in 0.1 m citrate‐phosphate buffer electrolyte at pH 3.8 (b,d); kinetic traces at 625 nm of NiO/P1 and NiO/P1‐MA e). The solid lines indicate fits from the target analysis.

To unravel the effect of co‐adsorbed MA on the photoinduced hole injection and recombination dynamics between NiO and P1, TA experiments have been carried out. **Figure** **6** shows the TA spectra at various time delays of NiO/P1 a) and NiO/P1‐MA b) in 0.1 m pH 3.8 citrate‐phosphate buffer electrolyte after 500 nm excitation. The negative signal is due to ground state bleach of the P1 dye, P1^*^ gives a strong absorbance band ≈550–560 nm^[^ ^38^ ^]^, and P1^•−^ formed after hole injection absorbs ≈610 nm.^[^ ^38^ ^]^ The red‐shift with time can thus be related to the hole injection dynamics, which process typically occurs biphasic, either within the TA instrumental response time of 100–150 fs or in 1–20 ps.^[^ ^38^ ^]^ The TA signals decay in time due to charge recombination. The spectrum of NiO/P1‐MA at 250 fs, well before vibrational relaxation and twisting (Figures S14–S19, Supporting Information), is ≈25 nm blue‐shifted compared to that of NiO/P1 and comparable to NiO/P1 in acetonitrile,^[^ ^41^ ^]^ suggesting less photoinduced hole injection <100–150 fs. This is supported by the gap between the kinetic traces at 527 and 560 nm of NiO/P1 and NiO/P1‐MA (Figures 6c,d). As the signal at 527 nm is mainly due to ground state bleach and the signal at 560 nm is also due to P1^*^, the larger gap for NiO/P1‐MA indicates a much slower hole injection. Further evidence is provided by the TRPL results shown in Figure S18 (Supporting Information). For NiO/P1 the PL quenching due to hole injection is stronger than for NiO/P1‐MA, with only weak PL beyond the instrumental time resolution of the streak camera. The slower hole injection might be caused by the hydrophobic nature of the long alkyl chain of MA, likely decreasing the quantity of OH^−^ on the NiO surface, as indicated by Figure S25 (Supporting Information). This interpretation is in line with our recent works in which we observed a dual role of surface OH^−^, slowing down both photoinduced hole injection and charge recombination^[^ ^41^ ^]^ and the bias‐dependent interface photodynamics.^[^ ^42^ ^]^ Figure 6e compares the kinetic traces at 625 nm, which signal is for NiO/P1 mainly due to P1^•−^, while for NiO/P1‐MA at early ps times also P1^*^ is likely to contribute due to the slower hole injection (Figure 6d vs c). NiO/P1‐MA initially shows a faster decay than NiO/P1, likely due to the P1^*^ decay. At later times the decay for NiO/P1‐MA is slower than for NiO/P1, indicating slower charge recombination and electron transfer from P1^•−^ to MA is unlikely. The photophysical models used for target analysis and the obtained species‐associated spectra and lifetimes are provided in Figure S19 (Supporting Information). The species‐associated spectra are blue‐shifted for NiO/P1‐MA compared to NiO/P1 as a result of inhibited twisting of P1^•−^ caused by the presence of MA.

### 2.3 Effect of Reduced Twisting on the Photoelectrochemical Performance

**Figure 7:**
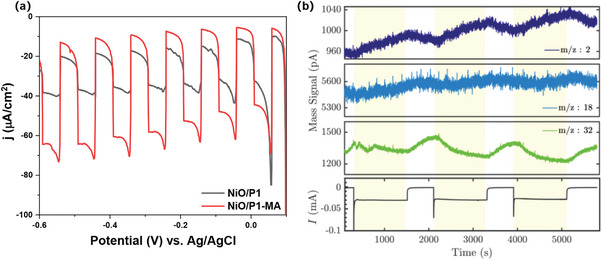
Photoelectrochemical performance in 0.1 m citrate‐phosphate buffer solution at pH 3.8 with chopped illumination with 1 sun a); EC‐MS results of NiO/P1‐MA at −0.35 V versus Ag/AgCl with 460 nm LED illumination b).

To evaluate how photoinduced twisting affects the photoelectrochemical performance, linear sweep voltammograms have been recorded in pH 3.8 citrate‐phosphate buffer solution, using chopped illumination (**Figure** **7**a). Before the measurements, the electrolyte was degassed by N_2_ for more than 20 min. to remove dissolved O_2_ and CO_2_. The lower dark current of ≈5 µA cm^−2^ can likely be explained by the reduced capacitance of NiO caused by the hydrophobic alkyl chains of the MA molecules. Despite the 25% lower light absorption efficiency (Figure S1, Supporting Information) and slower hole injection for NiO/P1‐MA compared to NiO/P1 as discussed above, NiO/P1‐MA shows a significantly larger photocurrent (Figures 7a). Very interestingly, illumination of the electrode with co‐adsorbed MA leads to bubble formation both on the cathode and the anode (Figures S20 and S21, Supporting Information). The composition of the bubbles was analyzed in a flow cell (flow rate 0.85 mL min^−1^, for details, see Figure S22, Supporting Information) using Electrochemical Mass Spectrometry (EC‐MS) and a 460 nm LED lamp for illumination. Due to the fast response (0.1 s.) of the EC‐MS system together with the low flow rates, we were able to analyze the composition of the gas evolved by the cathodic compartment of the photoelectrochemical cell. Figure 7b shows an increase in mass signal m/z = 2 representing H_2_ when starting illumination. The signal starts to decline as the light source is shut off. In addition, the m/z = 32 signal of O_2_ also shows a light response (a decay) upon illumination, indicating that O_2_ (likely formed on the anode and crossing over to the cathode compartment) is reduced by the cathode forming water. No sign of CO or CO_2_ is observed, which confirms that MA or dye decomposition do not significantly contribute to the photocurrents and H_2_ obtained. The formation of H_2_ by chemical transformation of the dye or MA is also not very likely in view of the following arguments. First, anchoring the MA onto the NiO via its carboxylic acid group causes deprotonation,^[^ ^43^ ^]^ and hence the only way to generate H_2_ from MA would be the reaction of water with ─CH_2_ or ─CH_3_ moieties. Such reaction is comparable to methane reforming, which produces hydrogen, but also CO or CO_2_ at the same time,^[^ ^44^ ^]^ which are not observed here. Second, the slower decays of the P1^*^ and P1^•−^ signals in the presence of MA (PL data Figure 5 and TA data Figure 6e) show that photoinduced electron transfer to MA is unlikely.

The P1 dye has been extensively studied for photocathodic applications and is generally accepted to be incapable of generating H_2_ in the absence of a H_2_ evolution catalyst.^[^ ^29^ ^]^ However, several studies exist that independently report H_2_ generation on various dye‐sensitized mesoporous NiO electrodes without the addition of any intended catalysts, under various conditions, though at comparatively low rates in all cases. These studies have been nicely summarized by Simonov et al., who proposed three possible mechanisms to explain the H_2_ evolution under the illumination of NiO/PMI‐6T‐TPA.^[^ ^45^, ^46^ ^]^ 1) The dye monolayer induces the H‐H bond formation; 2) Some metal cation impurities in the electrolyte are deposited on the surface through photoelectrochemical reduction, serving as catalysts for H_2_ evolution; 3) Some Ni dissolves (as Ni^2+^) and forms Ni nanoparticles deposited on loose, electrically disconnected NiO particles, which serve as active sites. The authors conclude that H_2_ generation likely predominately occurs via mechanism 3. Important differences between the present work and the work of Simonov et al. are that our mesoporous NiO likely does not contain ‘loose’ NiO particles, and NiO/P1 without MA does not have the ability to generate H_2_ under illumination. It is worth noting that Siminov et al. in their detailed study also modified the surface functionalized with PMI‐6T‐TPA by co‐adsorption of chenodeoxycholic acid–this reduced the performance of the photoelectrode for H_2_ evolution, explained by passivation of the NiO surface lowering the susceptibility for dissolution.

To understand which factors govern H_2_ generation on the NiO/P1‐MA, additional experiments were carried out. Photoelectrochemical studies in the presence of 15 vol% triethanolamine hole scavenger to suppress charge recombination again only show bubbles for NiO/P1‐MA (Figure S23 and Video S1, Supporting Information). We also like to reiterate that the generation of bubbles is only observed on p‐type semiconductors (NiO/P1‐MA and CuGaO_2_^[^ ^47^ ^]^/P1‐MA, Figure S26 and Video S2, Supporting Information), not on ZrO_2_/P1‐MA (Figure S27, Supporting Information). As previously stated, Siminov et al.^[^ ^46^ ^]^ assign the formation of Ni particles to the dissolution of Ni^2+^, proven by inductively coupled plasma mass spectrometry measurements, followed by reduction, which requires a reduction potential of 0.4 V versus RHE.^[^ ^46^ ^]^ The reason why only NiO/P1‐MA generates H_2_ under illumination might be that the inhibited twisting of the P1 D–A dye radical anion and resulting larger electrochemical potential are crucial to reduce Ni^2+^ into Ni nanoparticles acting as the catalytically active sites for H_2_ generation. Such dissolution, deposition, and catalysis have been previously proposed for nickel electrodes in acidic solutions.^[^ ^45^ ^]^ Moreover, Ni is a reasonably efficient catalyst for the hydrogen evolution reaction,^[^ ^46^ ^]^ and likely capable to accept electrons from the photoexcited dye. Evidence for such a mechanism is provided by the increase in Ni:O ratio after the photoelectrochemical performance studies of NiO/P1‐MA (Table S1, Supporting Information). It is also in line with work by Tian et al., who observed that NiO surface states can accept electrons and act as I_3_^−^ reduction catalysts.^[^ ^48^ ^]^ In summary, our work demonstrates the promise of TICT‐controlled dyes to realize photocathodes based on a straightforward design approach, not requiring any additional catalyst typically needed for H_2_ evolution.

## 3 Conclusion

Donor–acceptor (D–A) dyes have been designed to prolong light‐induced charge separation, by promoting the spatial separation of photoinduced electrons and holes via intramolecular charge transfer. We show that co‐adsorption of myristic acid (MA) onto NiO/P1 enables the production of H_2_, circumventing the need for an additional catalyst typically needed for H_2_ evolution. Time‐resolved photoluminescence and DFT studies show that twisting lowers the energy of the photoexcited P1 dye. Twisting is suppressed by the presence of co‐adsorbed MA, which slows down charge recombination as follows from fs transient absorption studies and increases the photocurrent. We assign the generation of H_2_ exclusively observed in the presence of co‐adsorbed MA to inhibited photoinduced twisting of the D–A dye radical anion, increasing the electrochemical potential of the electron to reduce the NiO surface, which in the reduced state can work as a catalytic site for H_2_ evolution. Our work illustrates the importance of understanding the effects of photoinduced twisting and demonstrates that control over the degree of twisting offers a simple design approach for efficient solar fuel devices.

## Conflict of Interest

The authors declare no conflict of interest.

## Supporting information

Supporting Information

Supplemental Video 1

Supplemental Video 2

## Data Availability

The data that support the findings of this study are available in the supplementary material of this article.
