# Supplementary material for: Limiting Molecular Twisting: Upgrading a Donor–Acceptor Dye to Drive H2 Evolution
Source: Adv Sci (Weinh). 2024 Aug 26;11(40):2403454. doi: 10.1002/advs.202403454 (PMC11516073; doi:10.1002/advs.202403454)
Supplement: Supplementary file 1 — Supporting Information [file ADVS-11-2403454-s003.pdf]

ADVANCED SCIENCE
Open Access

Supporting Information

for *Adv. Sci.*, DOI 10.1002/advs.202403454

Limiting Molecular Twisting: Upgrading a Donor–Acceptor Dye to Drive H_2 Evolution

*Kaijian Zhu, Ainoa Paradelo Rodríguez, Maria B. Brands, Titus de Haas, Francesco Buda, Joost N.H. Reek, Guido Mul and Annemarie Huijser**

Supporting information

Limitation of molecular twisting: Upgrading a donor-acceptor dye to drive H_2 evolution

Kaijian Zhu,[1] Ainoa Paradelo Rodríguez,[1] Maria B. Brands,[2] Titus de Haas,[3] Francesco Buda,[3] Joost N. H. Reek,[2] Guido Mul,[1] Annemarie Huijser [1]*

[1] PhotoCatalytic Synthesis Group, MESA+ Institute for Nanotechnology, University of Twente, P.O. Box 217, 7500 AE Enschede, the Netherlands.

[2] Van 't Hoff Institute for Molecular Sciences, Universiteit van Amsterdam, Science Park 904, 1098 XH Amsterdam, the Netherlands.

[3] Leiden Institute of Chemistry, Leiden University, P. O. Box 9502, 2300 RA Leiden, the Netherlands.

E-mail: j.m.huijser@utwente.nl

Experimental

Preparation of P1-sensitized NiO films

The NiO films were prepared by a chemical bath deposition method, followed by annealing. The 0.075 M $Ni(NO_3)_2{\bullet}6H_2O$ (Sigma-Aldrich, 99.999%), 0.2 M urea (Sigma-Aldrich, >99%) and 0.75 M ethanolamine (Sigma-Aldrich, >99%) precursors were sequentially dissolved into Milli-Q water. Cleaned fluorine-doped tin oxide (FTO) substrates purchased from Sigma-Aldrich were put into the solution, and films were grown on the FTO for 3 hours at 90 °C and washed by water. Finally, the NiO films were obtained by annealing at 450 °C for 1 hour in air. The NiO films were immersed into 0.3 mM P1 dye solution (4-(bis-4-[5-(2,2-dicyano-vinyl)-thiophene-2-yl]-phenyl-amino)-benzoic acid, Dyenamo, Sweden) in ethanol (Supelco, >99.9 %) with different concentrations of myristic acid (MA, Sigma-Aldrich, >98%, 0 mM, 3 mM, 15 mM and 40 mM) in the dark for ca. 16 hours.

ZrO_2 deposition

The cleaned FTO plates were treated in a UV-ozone photoreactor (UVP PR-100) for 30 min, after they were cut up in 20 × 20 mm pieces. Two sides of the plates were covered along the full length with adhesive tape (Scotch® Magic™ tape 8-1933R8), and ZrO_2 nanoparticle paste (Solaronix, ZT/SP Ref 46411) was applied on the top of the plate with a glass rod. The paste was then smeared from top to bottom using the edge of a glass plate, which was held at an acute angle (< 30°). This process was repeated until a homogeneous layer of the paste was obtained. The tape was removed and the plates were thermally annealed at 500 °C for 30 min (with a temperature ramp of 20 °C/min). The plates were taken out of the oven immediately after the program finished.

Characterization

X-ray diffraction (XRD, Bruker D2, Cu Kα source) and Scanning Electron Microscope (SEM, Zeiss MERLIN) were used to characterize the crystal structures, nanomorphology and thickness of the films. The valence states of the surface were characterized by X-ray Photoelectron Spectroscopy (XPS, PHI Quantera SXM). Raman spectra were recorded using an Avantes AvaRaman spectrometer and a 785 nm laser. The UV-Vis absorbance spectra of the films were recorded using a ThermoSci EVO600 spectrometer. The light absorption efficiency (F_A) is calculated according to the equation, with A the absorbance in optical density (OD):

$$F_A = (1 - 10^{-A}) \times 100\ \%$$

Time-resolved photoluminescence (TRPL)

The setup used for time-resolved photoluminescence has been described in detail in the previous work.[1-2] Briefly, a Fianium laser (FP-532-1-s, center wavelength 532 nm, pulse

duration of 300 fs, 80.37 MHz repetition rate) was used as a light souse. For experiments with UV excitation (267 nm), the output was focused into a 3 mm β-BaB_2O_4 crystal (Newlight Photonics). The residual 532 nm output was removed by three dichroic mirrors (Thorlabs, MBI-K04) and a FGUV11-UV filter (Thorlabs). The sample was kept in a quartz cuvette (Hellma, 10 mm optical path length) as the working electrode with 0.1 M phosphate buffer solution (PBS, pH=7) as the electrolyte, a Pt counter electrode and an Ag/AgCl reference electrode. The applied potential was controlled by a Emstat3 potentiostat (PalmSens). The spectral calibration was checked and adapted if necessary using a Hg/Ar calibration lamp (Oriel, LSP035).The spectral sensitivity of the PL spectra was corrected by the equation below, which was determined by measuring the spectrum of a black body lamp (Ocean Optics, HL-2000) with a calibrated spectrum:

$$Real\ PL\ spectrum = \frac{measured\ PL\ spectrum}{Y}$$

$$Y = 110 - 0.0012\ (wavelength\ in\ nm - 600)^2$$

The setup used for transient absorption experiments has been described in detail in our previous work.[1-2]

TDDFT calculations

All calculations were performed within the AMS2022.103 software package developed by SCM.[3] The P1 ground state geometry was optimized using the CAM-B3LYP functional[4] in an all-electron triple-ζ polarized (TZP) Slater-type basis set including relativistic effects by means of the scalar zeroth order regular approximation (ZORA).[5] The water solvation environment was accounted for by the implicit conductor like screening model (COSMO)[6-8] and dispersion corrections (D3) were included with Becke-Johnson damping.[9-11] The integration quality was improved over the default option by setting the numerical quality to

‘good’ and the SCF convergence criterium was tightened to 10e-8 Hartree/cycle. The optimized structure was verified to be a local minimum on the potential energy surface by performing a Hessian calculation (no imaginary frequencies). The excited state geometry optimization was performed within the framework of time-dependent density functional theory, using the Tamm-Dancoff approximation, at the same settings as the ground state optimization.[12] Each optimization step included four excitations from the ground state, while the P1 structure was minimized on the potential energy surface of the first excited state.

Photoelectrochemical (PEC) measurements

A VersaSTAT 3 Potentiostat was used to measure the photoelectrochemical (PEC) properties in a three-electrode cell under the illumination of a solar simulator with an AM 1.5G filter (Newport, 1 sun intensity) and a filter blocking UV light below 400 nm. An Ag/AgCl electrode and gold wire were used as the reference electrode and counter electrode. Prior to the measurements, the PBS (0.1 M)-citric acid (0.09 M) buffer electrolyte (pH around 3.8) was degassed by N_2 for more than 20 min. With a 5 mV/s scan rate, scans from high to low potential were carried out.

Operando photo-electrochemistry/mass-spectrometry (PEC/MS) measurements

The EC/MS equipment from SpectroInlets (Copenhagen, Denmark) and a 460 nm LED lamp (around 70 mW/cm^2) were used to conduct the PEC/MS measurements. The He was used as the carrier gas. Different m/z signals were recorded over time. A three-electrodes photoelectrochemical cell was used with Ag/AgCl (saturated reference electrode and Pt mesh counter electrode. A Nafion membrane was used to separate the reduction and oxidation reaction chambers. The electrolyte continues flow through the working electrode chamber and to the MS with the flow rate of 0.85 mL/min. Bio-Logic VSP potentiostat was used to applied the potential.

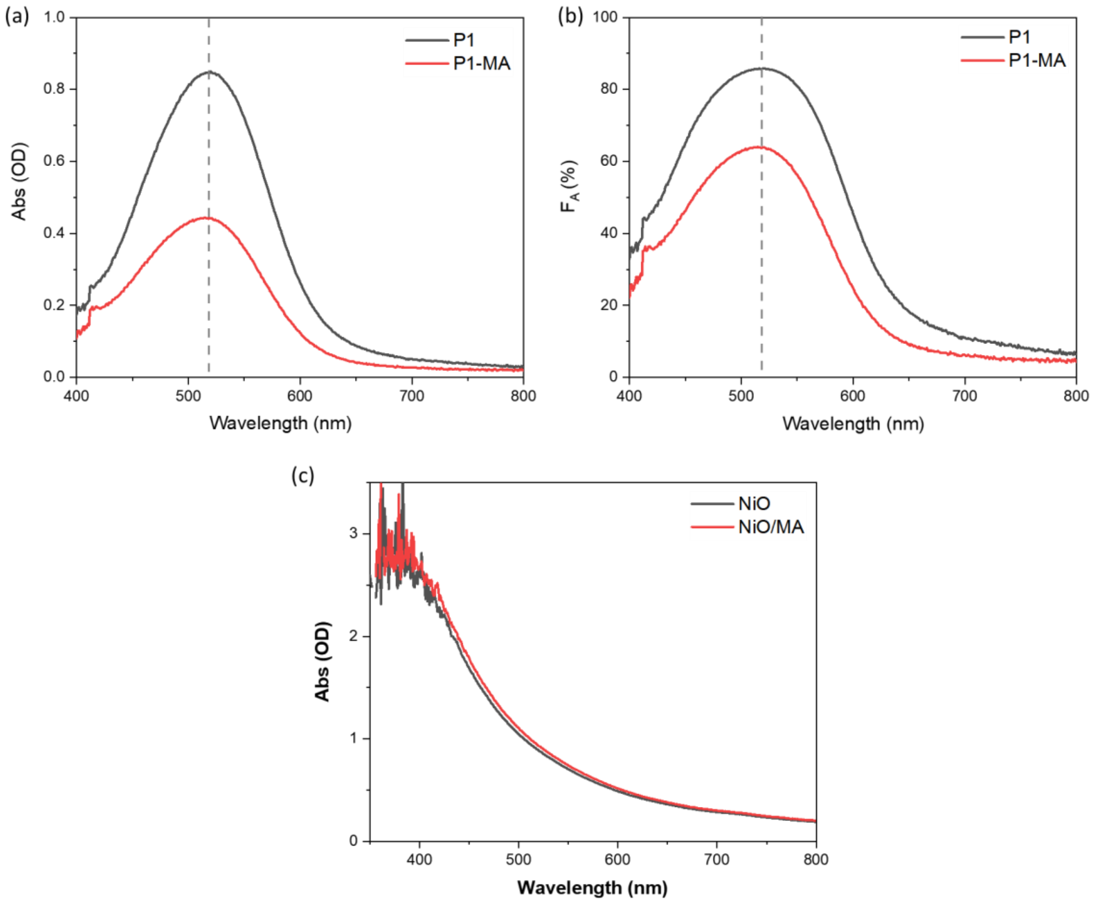

Figure S1. UV-Vis absorbance spectra (a) and light absorption efficiency (F_A) as function of wavelength (b) of NiO/P1 and NiO/P1-MA after subtracting the NiO signals. The dashed line indicates the maximum P1 absorption around 518 nm, both in the absence and presence of co-adsorbed MA. UV-vis spectra of NiO in the absence and presence of co-adsorbed MA without P1 dye (c).

The Raman spectra of NiO/P1 shown in Figure S2 are in agreement with the literature.[13] The most intense band around 1433 cm^{-1} is the symmetric ring stretching mode of a thiophene group. The bands around 1570 cm^{-1} are characteristic of C=C and C-C phenyl ring stretching. The band at 2224 cm^{-1} is due to the asymmetric nitril stretching mode of one of the maleonitrile acceptors.[13] Co-adsorption of MA does not shift these bands, showing that

interactions between P1 and MA are relatively weak, and the dye-NiO interactions are also not significantly altered by the MA.

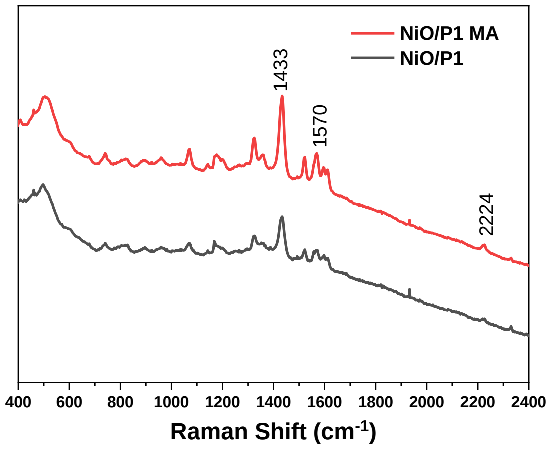

Figure S2. Raman spectra of NiO/P1 and NiO/P1-MA recorded using a 785 nm laser source.

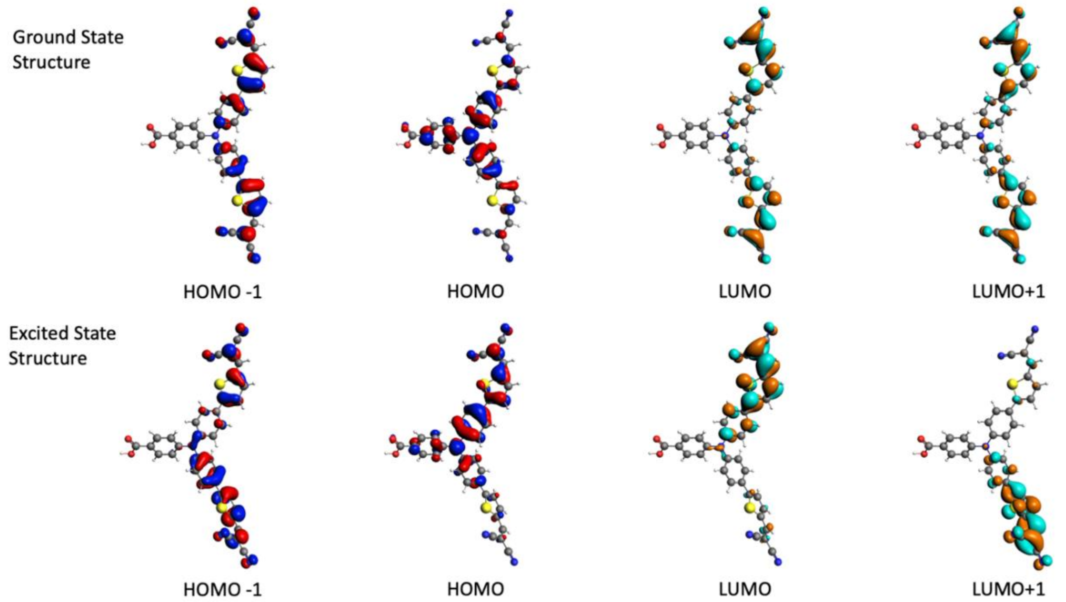

Figure S3. HOMO-1, HOMO, LUMO and LUMO+1 orbitals, calculated for the S_0 and S_1 relaxed geometries. Calculations were performed with the CAM-B3LYP functional in a TZP basis. The iso-surface value was set to 0.03.

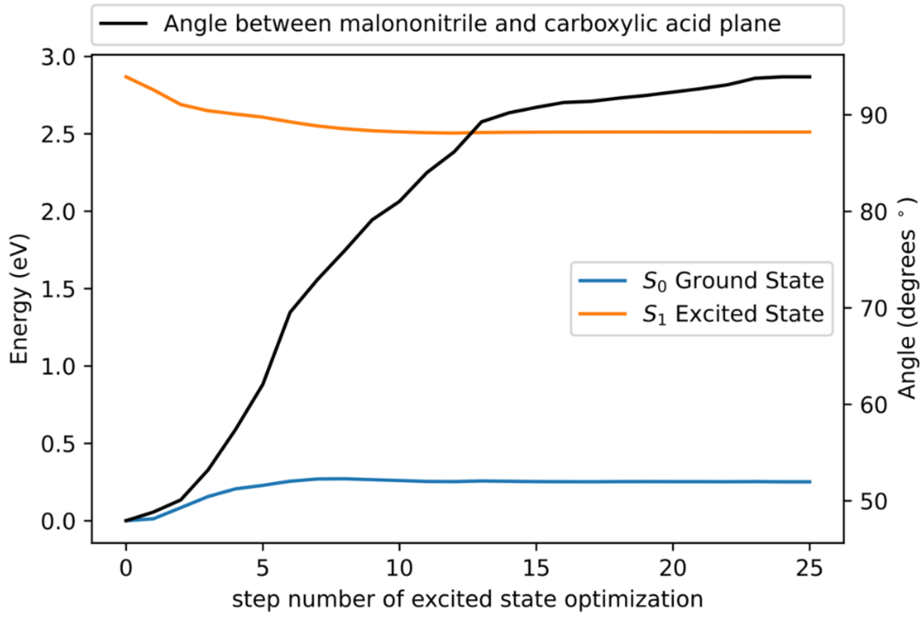

Figure S4. Ground state (blue) and excited state (orange) energies during a geometry optimization in the excited state, starting from the optimized ground state. During the relaxation, the angle between the planes of the malononitrile moiety and the carboxylic acid anchoring group increases from 47.9º to 93.9º. The excited state surface appears to be a simple decay curve without a barrier.

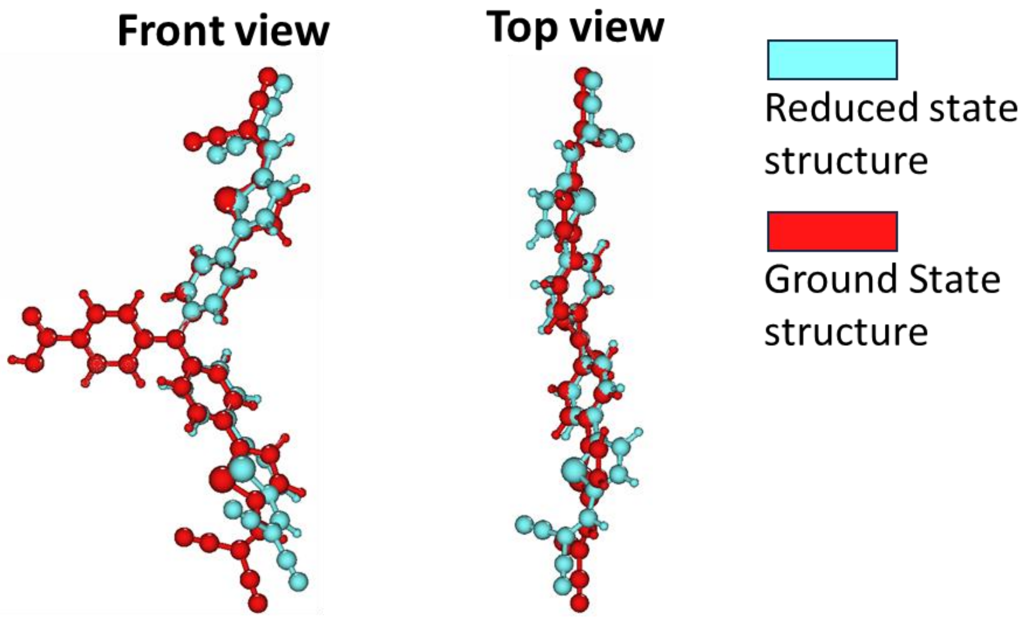

Figure S5. Structural rearrangement of P1 in the reduced state. The ground state optimized structure is visualized in red, while the reduced state optimized structure is depicted in cyan.

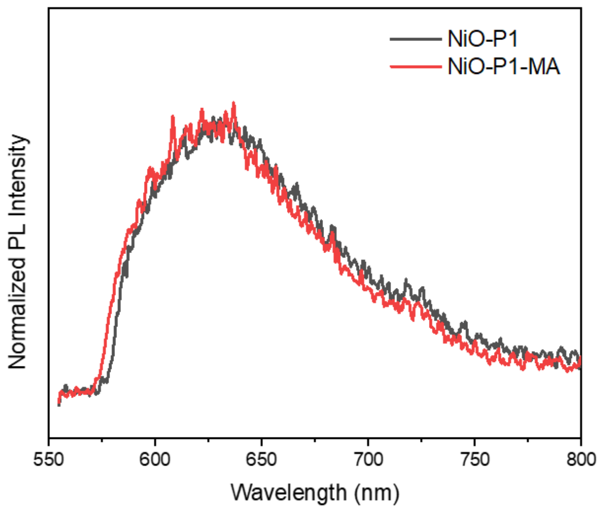

Figure S6. Steady-state photoluminescence spectra of NiO/P1 and NiO/P1-MA recorded by 532 nm excitation in air, detected using a 580 nm long-pass filter to block residual excitation light.

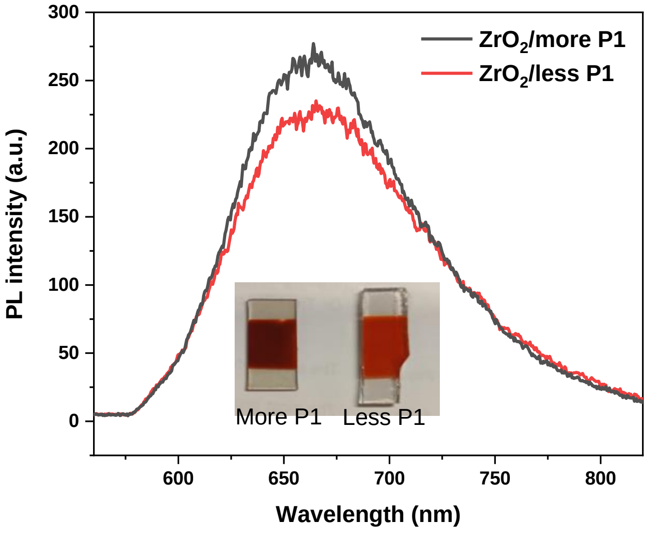

Figure S7. Steady-state photoluminescence spectra of ZrO_2 with low and high P1 dye loading.

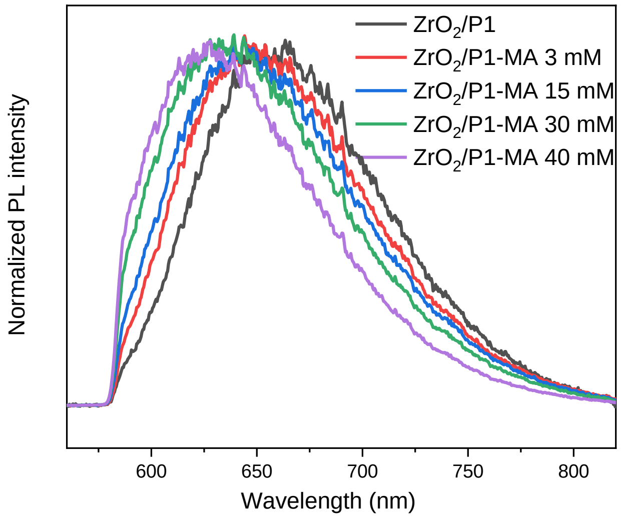

Figure S8. Steady-state photoluminescence spectra of ZrO_2/P1 prepared using different MA concentrations in the solution used for photosensitization.

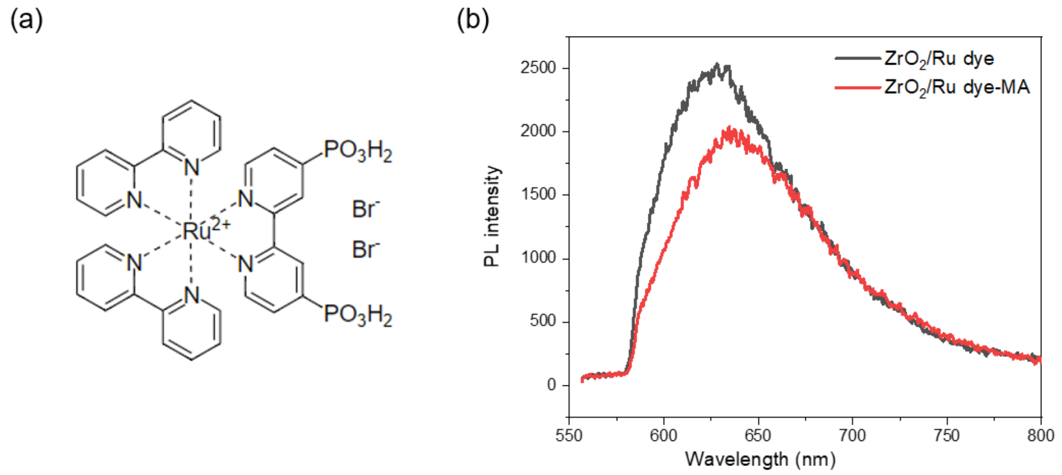

Figure S9. Ru dye molecular structure (a) and steady-state photoluminescence spectra of ZrO_2/Ru dye with and without co-adsorbed MA (40 mM in dipping solution) (b).

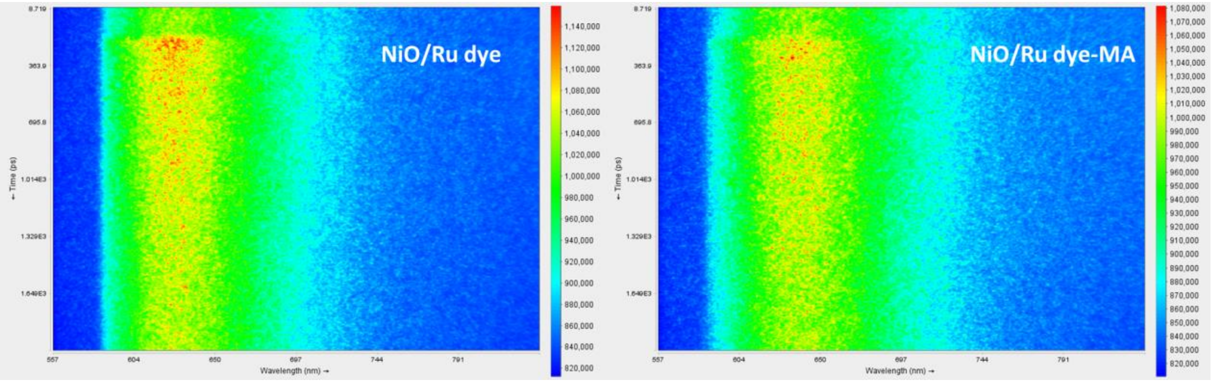

Figure S10. Time-resolved photoluminescence spectral profiles of ZrO_2/Ru dye (a) and ZrO_2/Ru dye-MA (b) in air after 532 nm excitation.

Figure S8 shows the steady-state photoluminescence spectra of ZrO_2/P1 and ZrO_2/P1-MA, showing a gradual blue-shift with increasing MA quantity. Figure S9 and S10 show the steady-state and time-resolved photoluminescence spectra of a Ru dye without D-π-A structure on ZrO_2 in the absence and presence of MA, not showing a blue-shift induced by MA alike observed for P1. The slightly red shift may be due to the different electronic coupling between the -COOH and -PO_3H_2 anchoring groups on the NiO. Figure S11 shows the steady-state photoluminescence spectra of ZrO_2/P1 prepared with 40 mM NA (a) and OA (b) present in the solution used for photosensitization. Co-adsorption of NA also induces a blue-shift, albeit less than MA, while the blue-shift due to OA is very small.

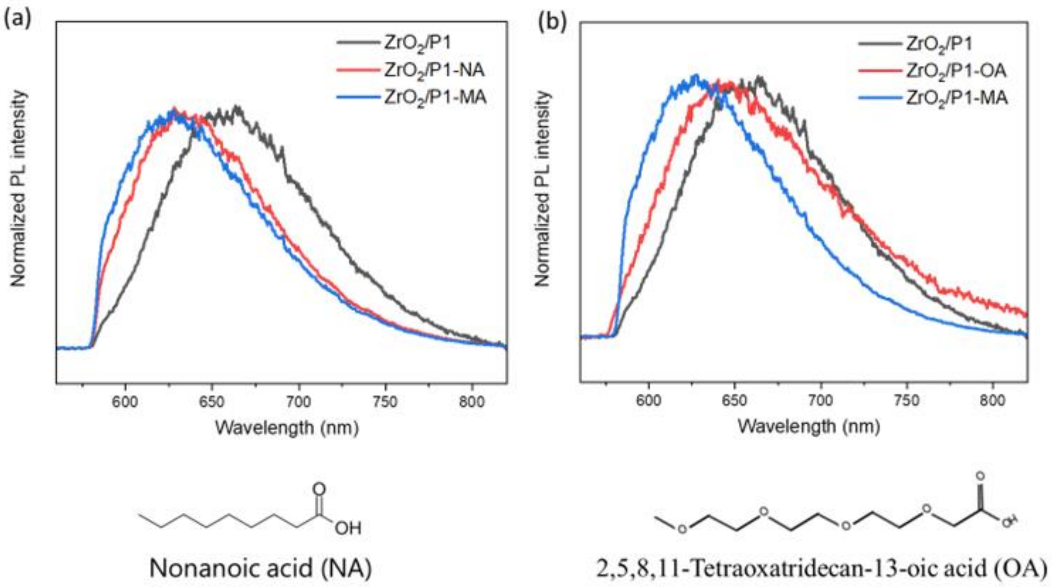

Figure S11. Steady-state photoluminescence spectra of ZrO_2/P1 prepared with 40 mM NA (a) and OA (b) in the solution used for photosensitization.

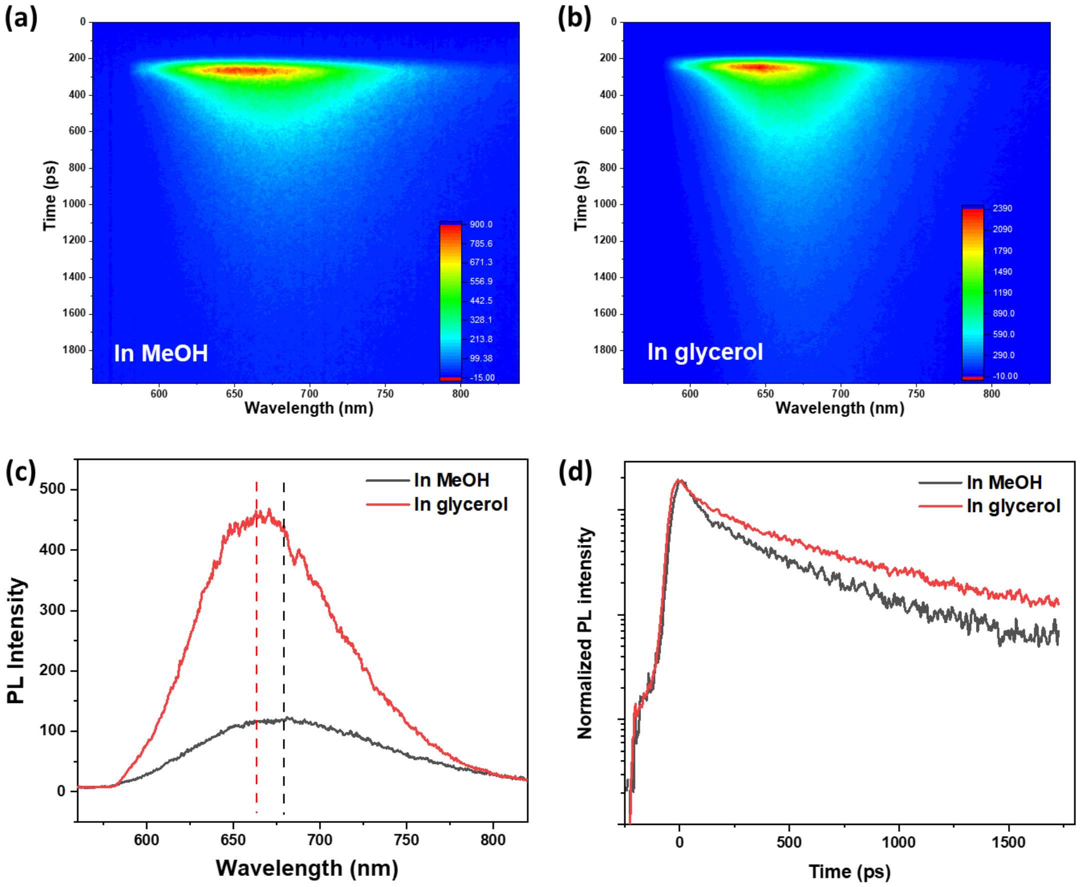

Figure S12. Time-resolved photoluminescence spectral profiles of ZrO_2/P1 in MeOH (a) and glycerol (b), recorded using 532 nm excitation; in (c) the steady-state photoluminescence spectra of ZrO_2/P1 in MeOH and glycerol; (d) presents the photoluminescence decay at 670 nm of ZrO_2/P1 and ZrO_2/P1-MA (d).

Figure S12 presents the time-resolved photoluminescence data of ZrO_2/P1 in MeOH or glycerol, both protic solvents, but with different viscosity. It is obvious that in glycerol, *i.e.* a higher viscosity, the PL spectra also show a blue-shift and higher intensity compared to in MeOH, alike observed with co-adsorption of MA (Figure 4 main text). Figure S13 shows the PL decays at indicated wavelengths of ZrO_2/P1 and ZrO_2/P1-MA, including fits from global analysis.

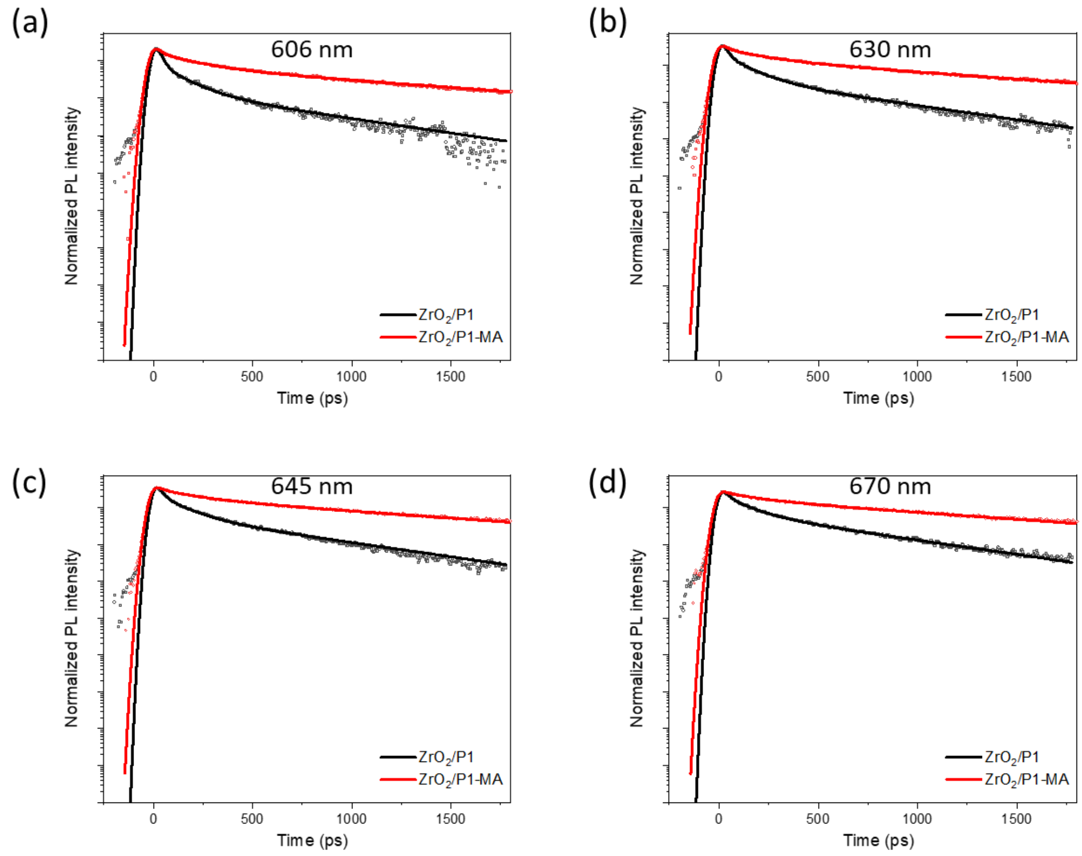

Figure S13. Photoluminescence decay at 606 nm (a), 630 nm (b), 645 nm (c) and 670 nm (d) of ZrO_2/P1 and ZrO_2/P1-MA. In all cases the sample was excited at 532 nm. The solid lines indicate fits from global analysis.

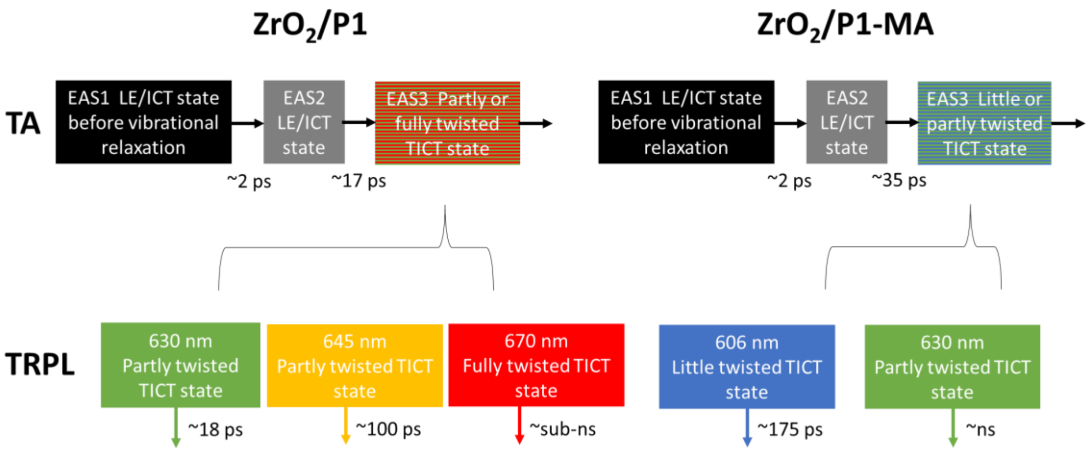

Figure S14. Photophysical models used for global analysis of the TA and TRPL data of ZrO_2/P1 and ZrO_2/P1-MA.

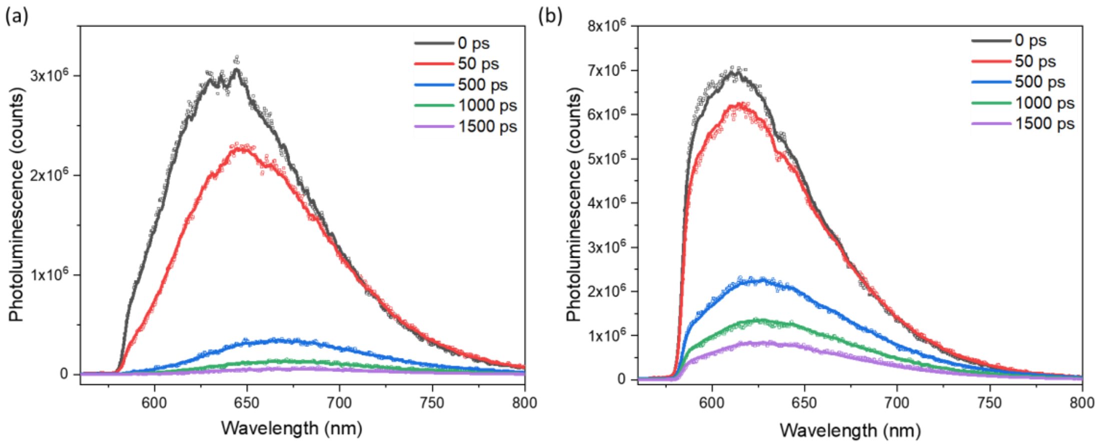

Figure S15. PL spectra of ZrO_2/P1 (a) and ZrO_2/P1-MA after 532 nm excitation in air. The solid lines indicate fits from global analysis. The most intense spectrum is defined as time 0.

Figure S14 shows the photophysical models used for global analysis of the TA and TRPL data of ZrO_2/P1 and ZrO_2/P1-MA. The spectra and fitting results are shown in Figure S15.

The data are well described by using the model in Figure S14. The evolution associated spectra (EAS) and associated time scales obtained from global analysis of the TA data are shown in Figure S16. EAS1 likely presents the LE/ICT state before vibrational relaxation occurring in ca. 2 ps, EAS2 the LE/ICT state after vibrational relaxation and EAS3 the (to some degree) twisted TICT state. We assign the PL bands centered at 606 nm, 630 nm and 665 nm to a little, partly and fully twisted TICT state, with the first only observed in presence of MA and the latter only in absence of MA. Figure S17 indicates the model fits the data well.

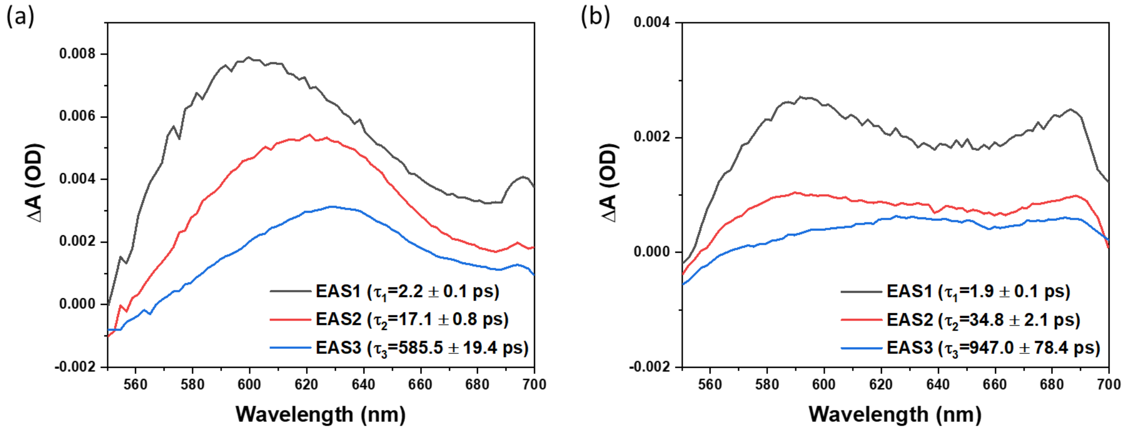

Figure S16. Evolution associated spectra (EAS) and corresponding lifetimes of ZrO_2/P1 (a) and ZrO_2/P1-MA (b), obtained from global analysis of the TA data using a sequential model.

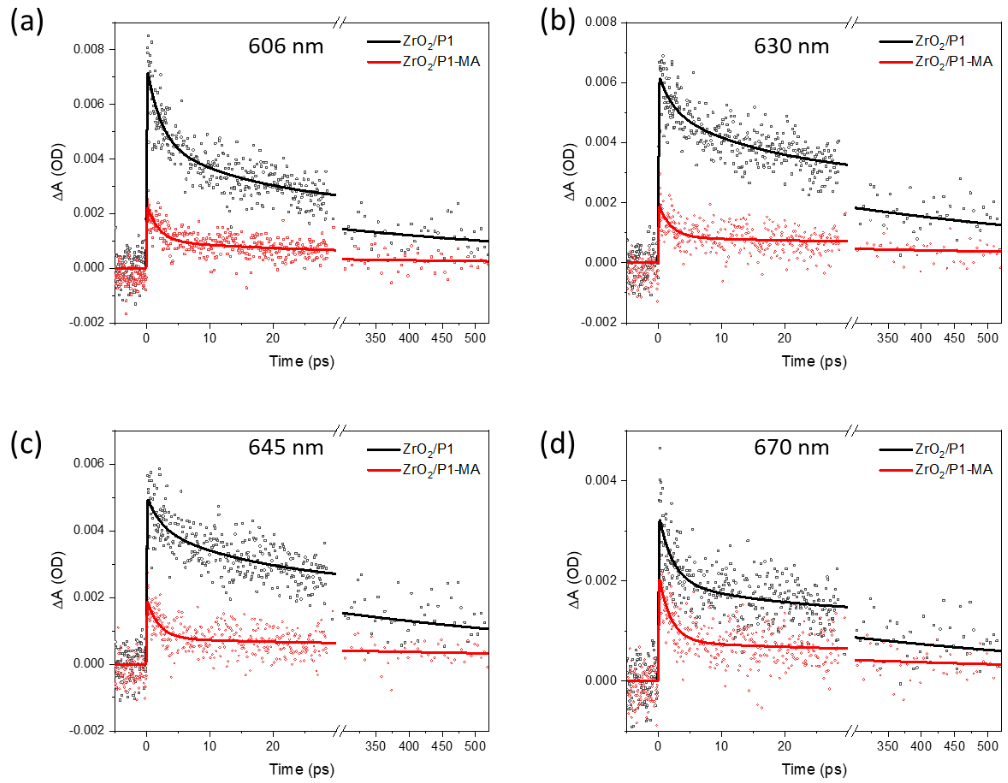

Figure S17. Kinetic traces at 606 nm (a), 630 nm (b), 645 nm (c) and 670 nm (d) of ZrO_2/P1 and ZrO_2/P1-MA. The solid lines indicate fits from global analysis.

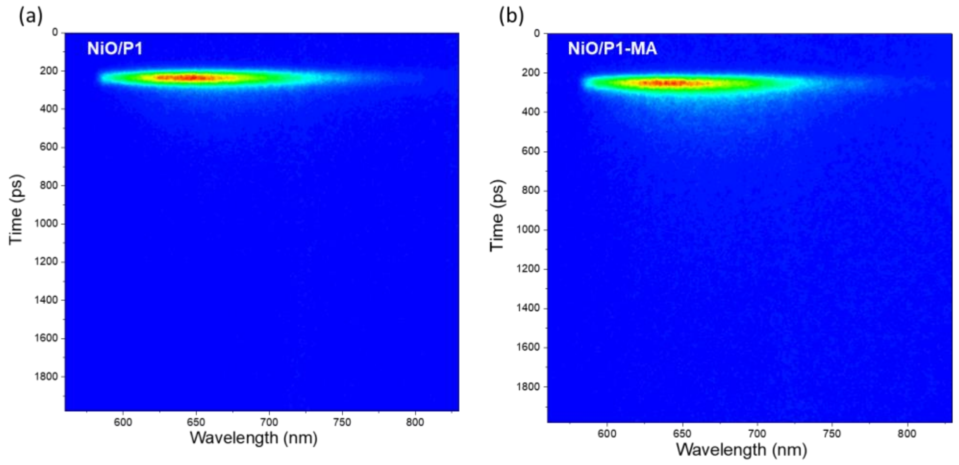

Figure S18. Time-resolved fluorescence spectral decay profiles of NiO/P1 (a) and NiO/P1-MA (b) in 0.1 M citrate-phosphate buffer electrolyte (pH = 3.8), obtained using 532 nm excitation.

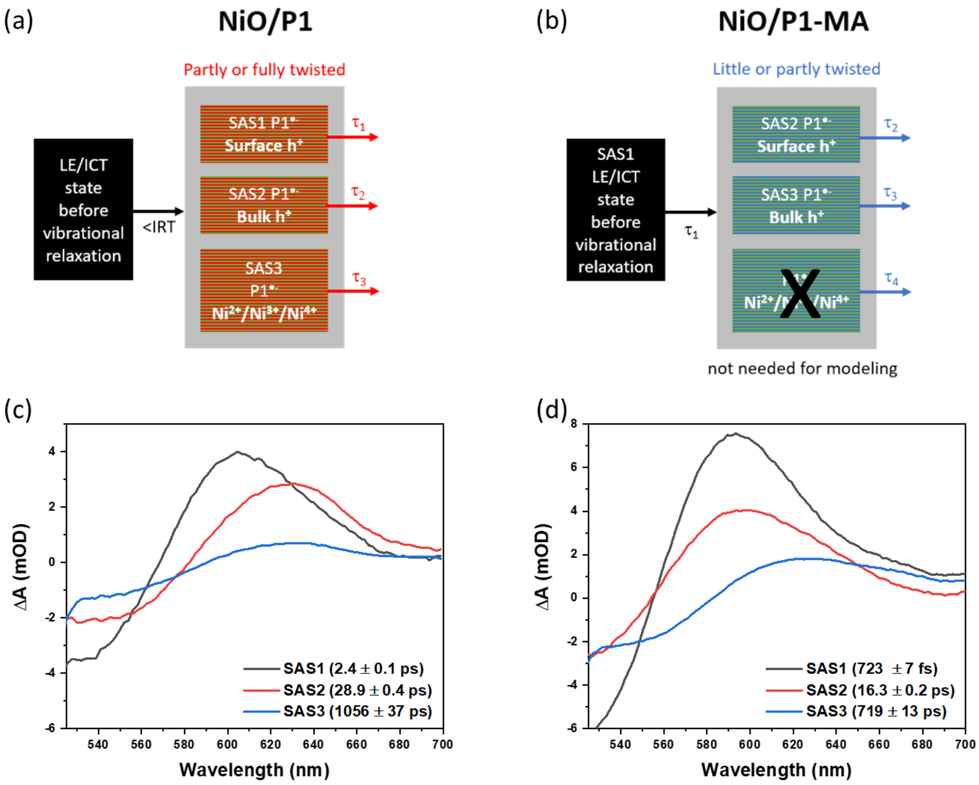

Figure S19. Photophysical models used for target analysis of the TA data of NiO/P1 (a) and NiO/P1-MA (b), taking into account different hole injection rates. IRT = instrumental response time (100-150 fs). Species associated spectra (SAS) and corresponding lifetimes obtained from target analysis for NiO/P1 (c) and NiO/P1-MA (d).

Figure S18 shows the time-resolved photoluminescence profiles of NiO/P1 and NiO/P1-MA, obtained using 532 nm excitation. Figure S19 shows the photophysical models introduced in our earlier work[2] used for target analysis of the TA data of NiO/P1 and NiO/P1-MA and the obtained species associated spectra and corresponding lifetimes. The models assumes hole injection from P1* into NiO to occur either within the instrumental response time (<IRT, 100-150 fs, for NiO/P1) or in several ps (for NiO/P1-MA, causing a blue-shifted TA spectrum compared to NiO/P1 at e.g. 250 fs, see Figs. 6a and 6b and main text), as the presence of MA clearly slows down hole injection as evident from the gap in signals at 527 nm and 560 nm (Figures 6c and 6d main text). Hole injection hence likely occurs prior to vibrational

relaxation in ca. 2 ps (Fig. S14), as also reported by Gustavsson and co-workers.[14] Several charge recombination rates are observed, which we assigned earlier to charge recombination between $P1^{\bullet-}$ and h^+ trapped at the NiO surface (the fastest recombination process) or more mobile h^+ capable to reach the NiO bulk (slower recombination). Also Ni^{2+}, Ni^{3+} and Ni^{4+} trap states may contribute to the signal.[15] Light-induced P1 twisting and the suppression thereof by MA may influence the TA spectrotemporal dynamics as well. The observation that after hole injection the TA spectrum of NiO/P1-MA is still blue-shifted relative to that of NiO/P1 (ca. 15 nm at 5 ps, Figs. 6 and 6b main text) may suggest that P1 twisting on NiO occurs faster than on ZrO_2 (Figure S16) due to charge separation with the NiO. Twisting has not been included in the modeling to avoid overfitting. For NiO/P1, charge recombination is assumed to occur between partly or fully twisted $P1^{\bullet-}$ with holes in the NiO. For NiO/P1-MA, charge recombination is assumed to occur between little or partly twisted $P1^{\bullet-}$ with holes in the NiO. Though these models are likely a simplification of the reality, they describe the TA data well.

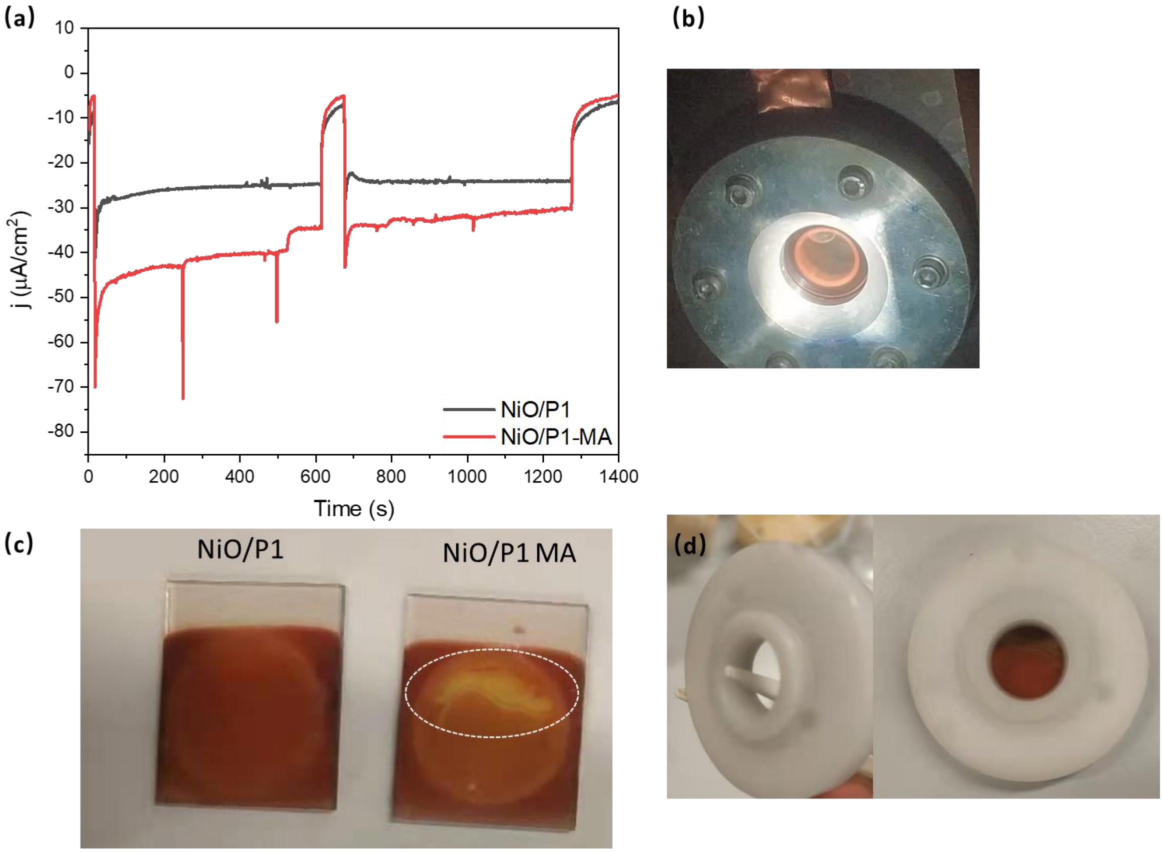

Figure S20. I-t curves at -0.35 V vs. Ag/AgCl of NiO/P1 and NiO/P1-MA in citric acid-phosphate buffer solution (pH = 3.8) with chopped illumination with 1 sun (AM 1.5G, >400 nm) (a); the cell used (b); digital photographs of NiO/P1 and NiO/P1-MA after the measurements (c); the sample holder (d).

Figure S20 shows the i-t curves of NiO/P1 and NiO/P1-MA measured in the cell shown in Figure S20b. The generation of bubbles causes dye and possibly also MA losses (Figure S20c), **when the bubbles could not easily escape** (Figure S20d shows the sample holder), likely also giving rise to the spikes in photocurrent observed in the presence of MA and the decrease in photocurrent (Figure S20a). However, it is obvious that the photocurrent for NiO/P1-MA is higher than for NiO/P1, in agreement with the linear sweep voltammetry results shown in Figure 7a in the main text. To further investigate the stability of NiO/P1-MA, the i-t curves were measured in a cell not blocking the bubbles (Figure S21a, b), showing a

stable photocurrent and without obvious dye losses even after long measurement time (Figure S21c). In this open cell in Figure S21, it is not accurate to quantify the surface area, because of capillary effects and exposed sides (some sample is formed on the side of the FTO during preparation). Therefore, here we only want to show the system is stable in this time range.

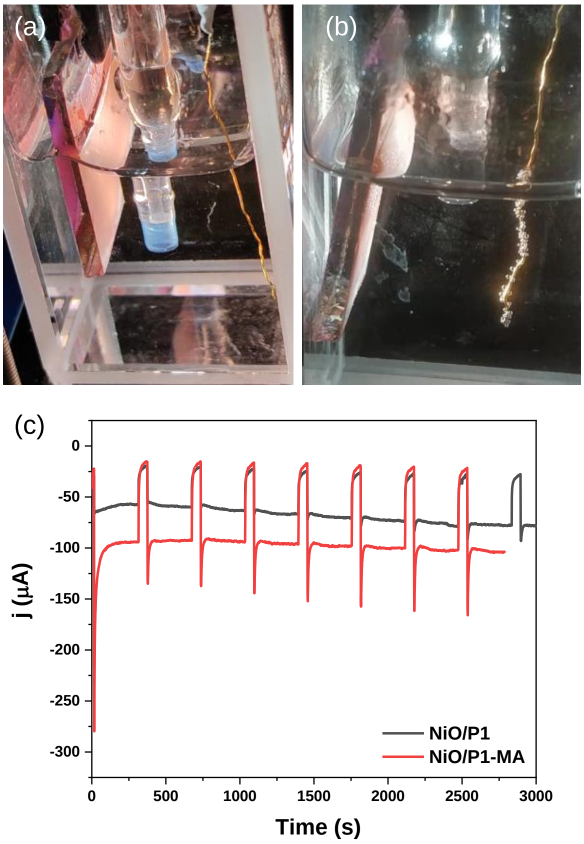

Figure S21. Digital photographs of NiO/P1 (a), NiO/P1-MA (b) and the corresponding photocurrent (c) during the i-t measurement at -0.35 V vs. Ag/AgCl in 0.1 M citrate-phosphate buffer solution at pH = 3.8 with chopped illumination with 1 sun (AM 1.5G, >400 nm).

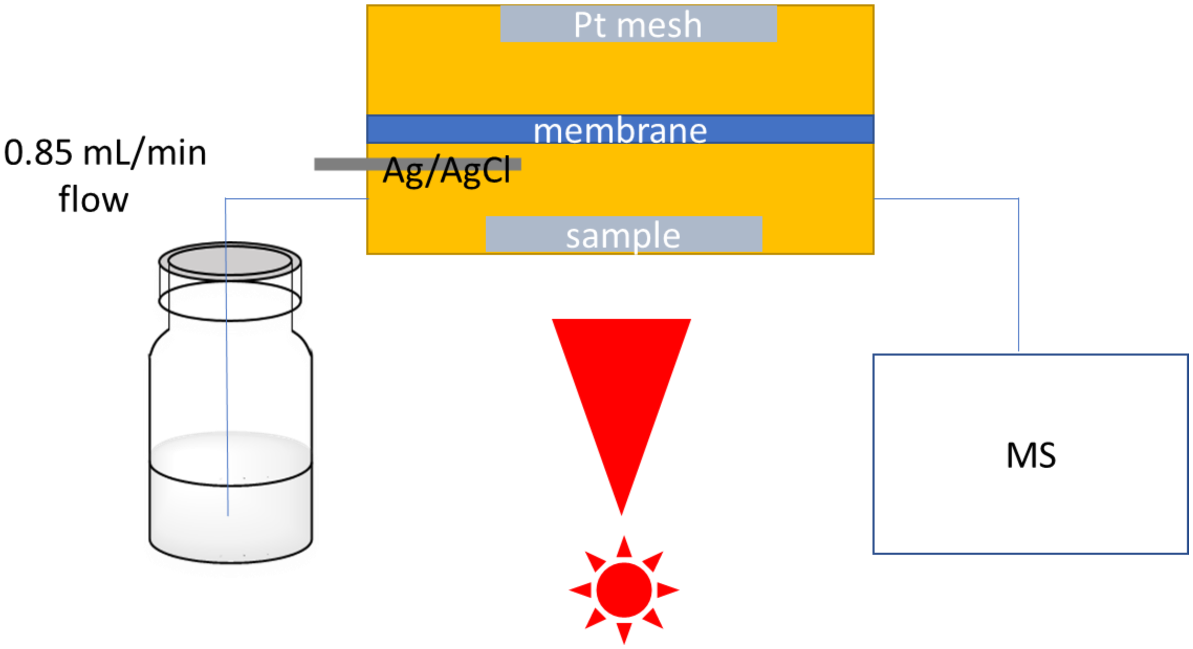

Figure S22. Schematic diagram of the home-assembled light source combined with electrochemical mass spectrometry (EC-MS) experiments.

Figure S22 shows a schematic diagram of the setup used for EC-MS experiments. The setup was stabilized by using a continuous He flow through the electrolyte. The home-designed three electrodes cell was put in the position where the sample is faced up to prevent bubble accumulation at the surface and dye losses. The electrolyte flow was kept as low as possible and the rate is around 0.85 mL/min.

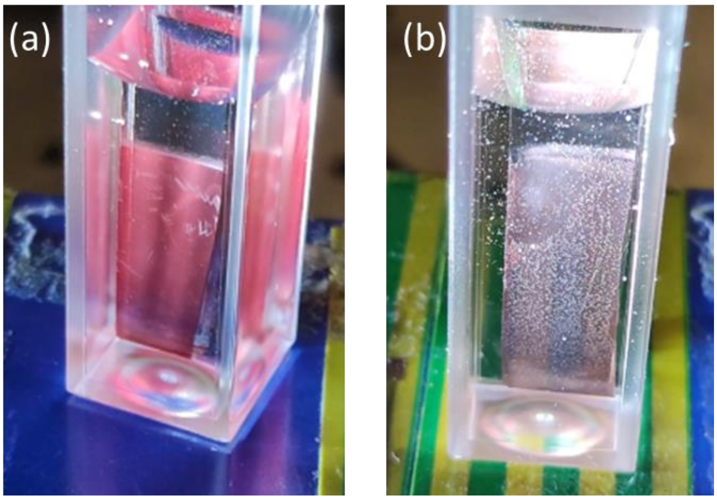

Figure S23. Digital photographs of NiO/P1 (a) and NiO/P1-MA (b) in 0.1 M citrate-phosphate buffer solution (pH = 3.8) with 15 vol% triethanolamine (TEOA) hole scavenger under illumination with 1 sun (AM 1.5G, >400 nm).

Figure S23 presents digital photographs of NiO/P1 (a) and NiO/P1-MA (b) in 0.1 M citrate-phosphate buffer solution (pH = 3.8) with 15 vol% triethanolamine (TEOA) hole scavenger under illumination with 1 sun (AM 1.5G, >400 nm), clearly showing bubble generation for the latter. Figure S24 shows the Ni $2p_{3/2}$ XPS spectra of NiO/P1 (a, c) and NiO/P1-MA (b, d) before and after the photoelectrochemical measurement, demonstrating no significant changes in Ni oxidation states. Figure S26 presents a digital photograph of $CuGaO_2$/P1-MA (b) in 0.1 M citrate-phosphate buffer solution with 15 vol% triethanolamine (TEOA) hole scavenger under illumination with 1 sun (AM 1.5G, >400 nm), also showing bubble formation.

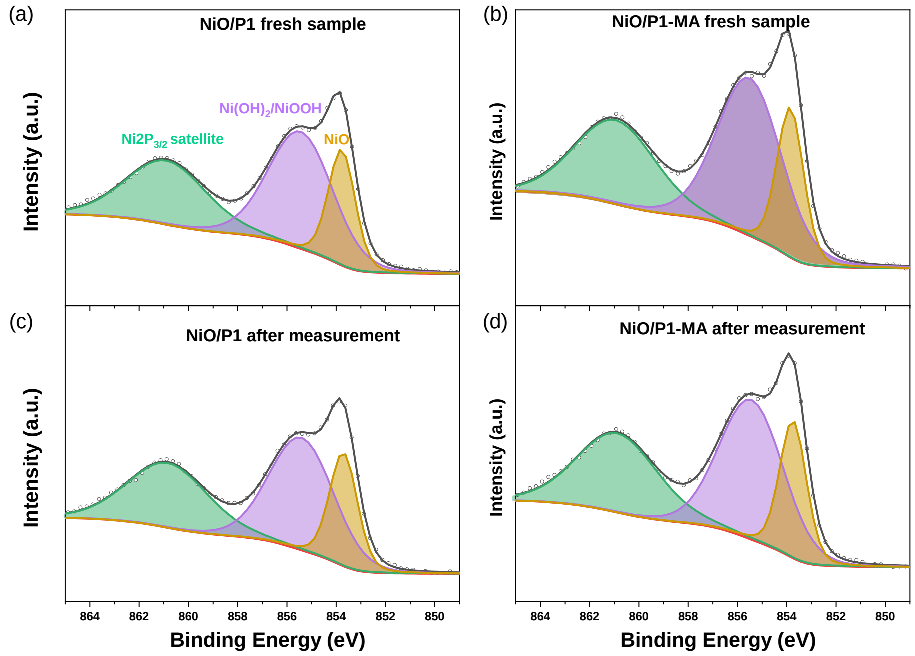

Figure S24. Ni $2p_{3/2}$ XPS spectra of NiO/P1 (a, c) and NiO/P1-MA (b, d) before and after the photoelectrochemical measurement.

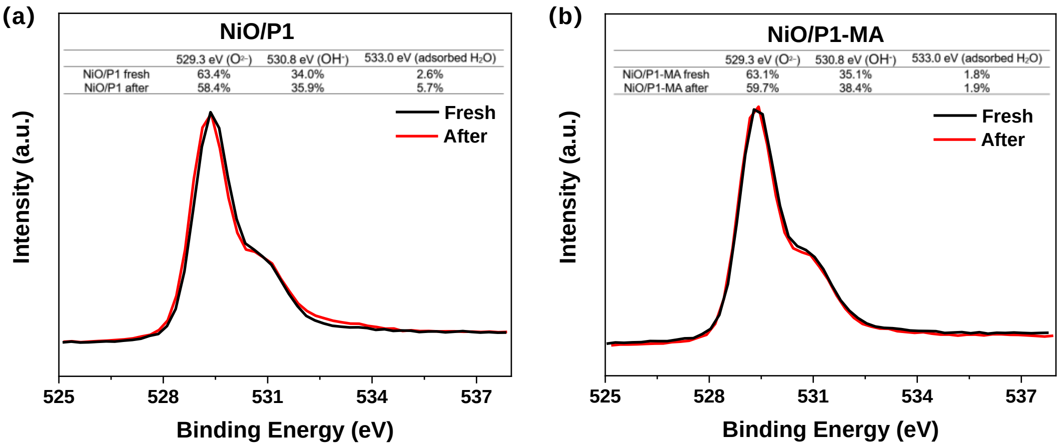

Figure S25. O1s XPS spectra of NiO/P1 (a) and NiO/P1-MA (b) before and after the photoelectrochemical measurement, with the insert presenting the results from peak deconvolution.

Table S1. The ratio of Ni:O before and after the photoelectrochemical measurement.

	C	O	Ni	Ni:O
NiO/P1				
Fresh	38.41	34.77	26.83	0.77
	40.30	34.71	24.99	0.72
After reaction	37.93	35.48	26.60	0.75
	37.61	34.78	27.61	0.79
NiO/P1-MA				
Fresh	35.04	37.03	27.93	0.75
	36.06	36.20	27.74	0.76
After reaction	30.38	37.79	31.83	0.84
	32.06	37.55	30.38	0.81

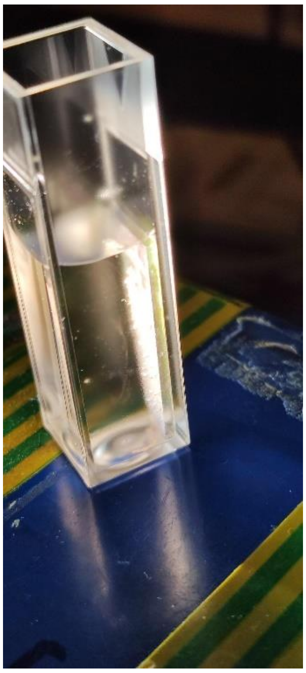

Figure S26. Digital photograph of $CuGaO_2$/P1-MA (b) in 0.1 M citrate-phosphate buffer solution with 15 vol% triethanolamine (TEOA) hole scavenger under illumination with 1 sun (AM 1.5G, >400 nm).

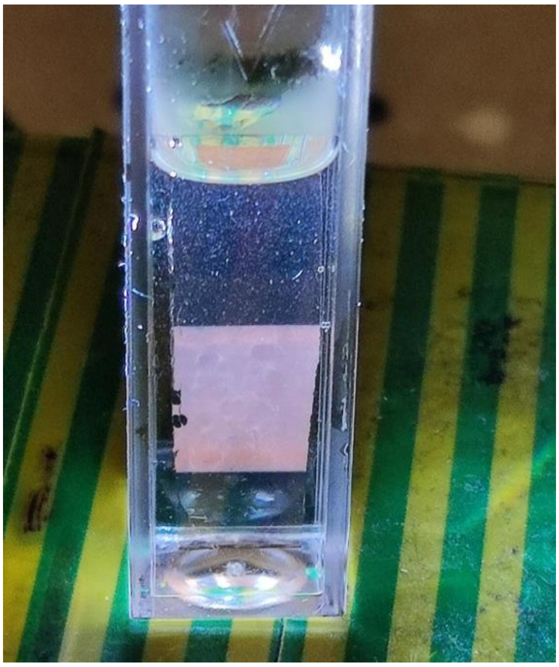

Figure S27. Digital photograph of ZrO_2/P1-MA (b) in 0.1 M citrate-phosphate buffer solution with 15 vol% triethanolamine (TEOA) hole scavenger under illumination with 1 sun (AM 1.5G, >400 nm).

References

[1] K. Zhu, S. K. Frehan, A. M. Jaros, D. B. O'Neill, J. P. Korterik, K. Wenderich, G. Mul, A. Huijser, *J. Phys. Chem. C* **2021**, *125*, 16049-16058.

[2] K. Zhu, S. K. Frehan, G. Mul, A. Huijser, *J. Am. Chem. Soc*. **2022**, *44*, 11010-1018.

[3] R. Rüger, M. Franchini, T. Trnka, A. Yakovlev, E. van Lenthe, P. Philipsen, T. van Vuren, B. Klumpers, T. Soini, Theor. Chem. **2021**.

[4] T. Yanai, D. P. Tew, N. C. Handy, *Chem. Phys. Lett.* **2004**, *393*, 51–57.

[5] E. Van Lenthe, *J. Chem. Phys.* **1999**, *110*, 8943–8953.

[6] A. Klamt, *J. Phys. Chem.* **1995**, *99*, 2224–2235.

[7] A. Klamt, V. Jonas, *J. Chem. Phys.* **1996**, *105*, 9972–9981.

[8] C. C. Pye, T. Ziegler, E. Van Lenthe, J. N. Louwen, *Can. J. Chem* **2009**, *87*, 790–797.

[9] S. Grimme, *J. Comput. Chem.* **2004**, *25*, 1463–1473.

[10] S. Grimme, J. Antony, S. Ehrlich, H. Krieg, *The Journal of Chemical Physics* **2010**, *132*, 154104.

[11] S. Grimme, S. Ehrlich, L. Goerigk, *Journal of Computational Chemistry* **2011**, *32*, 1456–1465.

[12] S. Hirata, M. Head-Gordon, *Chem. Phys. Lett.* **1999**, *314*, 291–299.

[13] G.H. Summers, G. Lowe, J. Lefebvre, S. Ngwerume, M. Bräutigam, B. Dietzek, J.E. Camp, E. A. Gibson, *ChemPhysChem* **2017**, *18*, 406 – 414.

[14] V. Maffeis, B. Jousselme, T. Gustavsson, *Photoch. Photobio. Sci.* **2021**, *20*, 1257-1271.

[15] L. D'Amario, J. Föhlinger, G. Boschloo, L. Hammarström, *Chem. Sci.* **2018**, *9*, 223-230.
